# Age-specific risks, severity, time course, and outcome of bleeding on long-term antiplatelet treatment after vascular events: a population-based cohort study

**DOI:** 10.1016/S0140-6736(17)30770-5

**Published:** 2017-07-29

**Authors:** Linxin Li, Olivia C Geraghty, Ziyah Mehta, Peter M Rothwell, Oxford Vascular Study

**Affiliations:** a Centre for Prevention of Stroke and Dementia, Nuffield Department of Clinical Neurosciences, John Radcliffe Hospital, University of Oxford, Oxford, UK

## Abstract

**Background:**

Lifelong antiplatelet treatment is recommended after ischaemic vascular events, on the basis of trials done mainly in patients younger than 75 years. Upper gastrointestinal bleeding is a serious complication, but had low case fatality in trials of aspirin and is not generally thought to cause long-term disability. Consequently, although co-prescription of proton-pump inhibitors (PPIs) reduces upper gastrointestinal bleeds by 70–90%, uptake is low and guidelines are conflicting. We aimed to assess the risk, time course, and outcomes of bleeding on antiplatelet treatment for secondary prevention in patients of all ages.

**Methods:**

We did a prospective population-based cohort study in patients with a first transient ischaemic attack, ischaemic stroke, or myocardial infarction treated with antiplatelet drugs (mainly aspirin based, without routine PPI use) after the event in the Oxford Vascular Study from 2002 to 2012, with follow-up until 2013. We determined type, severity, outcome (disability or death), and time course of bleeding requiring medical attention by face-to-face follow-up for 10 years. We estimated age-specific numbers needed to treat (NNT) to prevent upper gastrointestinal bleeding with routine PPI co-prescription on the basis of Kaplan–Meier risk estimates and relative risk reduction estimates from previous trials.

**Findings:**

3166 patients (1582 [50%] aged ≥75 years) had 405 first bleeding events (n=218 gastrointestinal, n=45 intracranial, and n=142 other) during 13 509 patient-years of follow-up. Of the 314 patients (78%) with bleeds admitted to hospital, 117 (37%) were missed by administrative coding. Risk of non-major bleeding was unrelated to age, but major bleeding increased steeply with age (≥75 years hazard ratio [HR] 3·10, 95% CI 2·27–4·24; p<0·0001), particularly for fatal bleeds (5·53, 2·65–11·54; p<0·0001), and was sustained during long-term follow-up. The same was true of major upper gastrointestinal bleeds (≥75 years HR 4·13, 2·60–6·57; p<0·0001), particularly if disabling or fatal (10·26, 4·37–24·13; p<0·0001). At age 75 years or older, major upper gastrointestinal bleeds were mostly disabling or fatal (45 [62%] of 73 patients *vs* 101 [47%] of 213 patients with recurrent ischaemic stroke), and outnumbered disabling or fatal intracerebral haemorrhage (n=45 *vs* n=18), with an absolute risk of 9·15 (95% CI 6·67–12·24) per 1000 patient-years. The estimated NNT for routine PPI use to prevent one disabling or fatal upper gastrointestinal bleed over 5 years fell from 338 for individuals younger than 65 years, to 25 for individuals aged 85 years or older.

**Interpretation:**

In patients receiving aspirin-based antiplatelet treatment without routine PPI use, the long-term risk of major bleeding is higher and more sustained in older patients in practice than in the younger patients in previous trials, with a substantial risk of disabling or fatal upper gastrointestinal bleeding. Given that half of the major bleeds in patients aged 75 years or older were upper gastrointestinal, the estimated NNT for routine PPI use to prevent such bleeds is low, and co-prescription should be encouraged.

**Funding:**

Wellcome Trust, Wolfson Foundation, British Heart Foundation, Dunhill Medical Trust, National Institute of Health Research (NIHR), and the NIHR Oxford Biomedical Research Centre.

## Introduction

Roughly 40–66% of adults aged 75 years or older in the USA and Europe take daily aspirin or other antiplatelet drugs,^1^, ^2^ about half for secondary prevention of vascular disease, consistent with guideline recommendations for lifelong treatment.^3^, ^4^ Antiplatelet drugs increase the risk of major bleeding, particularly upper gastrointestinal bleeds,^5^ but this risk is reduced by 70–90% by proton-pump inhibitors (PPIs; trials are summarised in the appendix [p 2]).^6^ However, co-prescription of PPIs is not routine because of concerns about adverse effects,^7^, ^8^, ^9^, ^10^ and perhaps because upper gastrointestinal bleeds had a low case fatality in trials of aspirin^11^ and are not generally thought to cause permanent disability. Clinical guidelines on secondary prevention of vascular events make no recommendations on PPI use^3^, ^4^ and, although some consensus statements advocate use of these drugs in high-risk patients,^12^ definitions of high risk vary and uptake in practice remains low.^9^, ^10^

**Research in context**

**Evidence before this study**

Lifelong antiplatelet treatment is recommended after ischaemic vascular events on the basis of trials done at younger ages (mainly <75 years). Bleeding is a serious complication, but is most commonly upper gastrointestinal, which has had low case fatality in previous trials and is not generally thought to cause long-term disability. Consequently, although proton-pump inhibitors (PPIs) reduce upper gastrointestinal bleeds by 70–90%, uptake in clinical practice is low and guidelines on secondary prevention of vascular events make no recommendations on PPI use. Although some consensus statements have recommended co-prescription for high-risk patients, definitions of high risk vary. Yet little is known about the risk, time course, or functional outcomes of upper gastrointestinal bleeding at older ages (≥75 years) in routine clinical practice. We searched PubMed with the terms “antiplatelet and secondary prevention”, “antiplatelet and bleeding”, “aspirin and bleeding”, “GI bleeding”, “gastric protection and GI bleeding”, and “age and bleeding” for articles published in English before Jan 1, 2017. Published estimates of age-specific risks of bleeding vary by more than ten fold, particularly at older ages, and derive mainly from primary prevention settings, with reliance on administrative coding data only. We found no published data for the long-term time course of bleeding risk in older patients in secondary prevention, and no data for the functional outcome of upper gastrointestinal bleeding. Although the risk of upper gastrointestinal bleeding on antiplatelet treatment increases with age, it is uncertain whether older age alone is a sufficient indicator of high risk and hence routine co-prescription of PPIs.

**Added value of this study**

The findings of our large prospective population-based study of long-term antiplatelet treatment in secondary prevention of vascular disease show that the severity, case fatality, and poor functional outcome of bleeds increase with age. Moreover, in contrast with the general impression that upper gastrointestinal bleeds are mostly non-disabling with low case fatality, we showed that in patients aged 75 years or older, most major upper gastrointestinal bleeds were disabling or fatal, substantially outnumbering disabling or fatal intracerebral haemorrhage. Finally, we provided estimates of the likely effect of routine PPI use in patients aged 75 years or older on prevention of major upper gastrointestinal bleeds.

**Implications of all the available evidence**

In patients receiving secondary prevention with aspirin-based antiplatelet treatment without routine PPI use, the long-term risk of bleeding at age 75 years or older is much higher and more sustained than in the younger age groups included in previous trials, with particularly high risks of disabling or fatal upper gastrointestinal bleeding. Given that half of the major bleeds in patients aged 75 years or older were upper gastrointestinal, the estimated numbers needed to treat for routine PPI use to prevent major upper gastrointestinal bleed are low, and should be considered in future secondary prevention guidelines.

Because the absolute benefit of routine PPI use will depend mainly on the risk of upper gastrointestinal bleeding on long-term follow-up, and about half of patients taking antiplatelet drugs for secondary prevention are now aged 75 years or older (appendix pp 3–5), we need reliable estimates of age-specific risks and consequences of bleeding in a real-world setting. The risk of upper gastrointestinal bleeding on antiplatelet treatment increases with age,^13^, ^14^, ^15^ but it is uncertain whether older age alone is a sufficient indicator of high risk to justify routine co-prescription of PPIs. Published estimates of age-specific risks of bleeding vary by more than ten fold, particularly at older ages, and derive mainly from primary prevention settings,^16^ with relatively short follow-up.^13^, ^14^, ^15^, ^16^ Completeness of ascertainment of bleeding events is also uncertain in many studies because of a reliance on only administrative coding data. Previous trials (appendix pp 3–5) of antiplatelet drugs with face-to-face follow-up probably have better ascertainment, but recruited few patients aged 75 years or older, tended to exclude high-risk patients, and had relatively short follow-up times. Although the excess risk of bleeding attributable to aspirin declined after several years of follow-up in trials of primary prevention,^11^ the time course of bleeding risk in older patients receiving treatment for secondary prevention is uncertain and data for the functional outcome of upper gastrointestinal bleeds are scarce.

We aimed to determine the age-specific risks, site, severity, outcomes, time course, and predictors of bleeding complications in secondary prevention of vascular events, to compare the risks with those of recurrent ischaemic events and those reported in previous randomised trials, and to estimate the potential effect of routine PPI use on reducing bleeding.

## Methods

### Study design and participants

We did a population-based cohort study in consecutive patients who were first in the study period to have acute transient ischaemic attack, ischaemic stroke, or myocardial infarction, and were treated with antiplatelet drugs (ie, started anew or continued) in the Oxford Vascular Study (OXVASC) from 2002 to 2012, with follow-up until 2013. OXVASC is a population-based study of the incidence and outcome of all acute vascular events in a population of 92 728 individuals, irrespective of age, registered with 100 general practitioners in nine general practices in Oxfordshire, UK. The definitions of vascular events and the multiple overlapping methods used to achieve near complete ascertainment of all individuals with transient ischaemic attack, stroke, or myocardial infarction are detailed in the appendix (pp 34, 35) and have been reported previously.^17^

We excluded patients who started or continued oral anticoagulants after an event, but included those receiving premorbid oral anticoagulants who were switched to antiplatelet therapy after the event. Patients who took anticoagulants during subsequent follow-up were censored at the time of starting permanent anticoagulation. We excluded patients who were not given antithrombotic drugs because of recent bleeding, coagulation disorders, known allergy, other known bleeding tendency, or a decision for palliative care only. Written informed consent, or assent from relatives, was obtained from all participants, and OXVASC has been approved by the local ethics committee.

### Procedures

Demographics and vascular risk factors were obtained at initial assessment, as were risk factors for bleeding, including alcohol use, anaemia, history of peptic ulcer, renal failure, chronic liver disease, history of cancer, and weight. All medications taken before the event, at discharge, and at follow-up were recorded.

In patients with transient ischaemic attack and ischaemic stroke, long-term recommended antiplatelet treatment was aspirin (75 mg daily) plus dipyridamole (200 mg twice daily). In patients seen within 48 h of their acute event, or those seen later who were at high early risk of recurrent stroke (eg, ABCD^2^ score ≥4), initial treatment was with aspirin plus clopidogrel (75 mg daily) for 30 days. In patients with myocardial infarction, standard treatment was with aspirin plus clopidogrel for 6–12 months, followed by aspirin alone. No PPI or other gastric protection strategies were routinely co-prescribed. Brain imaging was required before initiation of antiplatelet treatment after stroke.

Patients were followed up face to face at 30 days, 6 months, and years 1, 5, and 10 by a study nurse or physician. Recurrent ischaemic events, bleeding events, and disability (modified Rankin Scale)^18^ were recorded at each follow-up visit. Follow-up was done via a carer in patients with dementia, and by telephone in patients who had moved out of the study area. Bleeding events were also identified by daily searches of all hospital admissions,^17^ by review of administrative diagnostic codes from hospital and primary care records, and by regular searches of blood transfusion records. All deaths (with causes) during follow-up were also recorded from death certificates and coroners' reports.^17^ We included only bleeding events for which the patient had sought medical attention or that were fatal before attention being sought. We excluded minor bleeds, such as bruising, that had not necessitated medical attention. We also excluded bleeds secondary to major trauma, major surgical procedures, or haematological malignancy.

Site of bleeding was classified as intracranial (intracerebral, subdural, or subarachnoid) and extracranial (upper gastrointestinal, lower gastrointestinal, epistaxis, genitourinary, and other). Cases of melaena without investigation or with normal investigations were classified as upper gastrointestinal. We used the Clopidogrel in Unstable angina to prevent Recurrent Events (CURE) criteria (appendix p 37)^19^ to define bleeding events as major and life-threatening or fatal. Bleeding events that required medical attention but did not fulfil the criteria for major bleed were defined as significant non-major bleeds. Bleeds were defined as disabling if they resulted in a deterioration in functional independence (modified Rankin Scale increased to ≥3, or increased by ≥1 point if premorbid modified Rankin Scale ≥3) at hospital discharge without recovery by the next follow-up visit. The cause of disability was coded, when possible, and included complications triggered directly by the bleed, such as myocardial infarction, ischaemic stroke, or heart failure.

### Statistical analyses

We derived estimates of risk from Kaplan–Meier analyses. In patients who had multiple bleeding events of the same severity, the first event was classified as the endpoint, irrespective of any difference in the type of events. However, in analysis of risk of more serious bleeds (ie, major bleeds, life-threatening or fatal bleeds, disabling or fatal bleeds) patients were not censored at the time of any preceding significant non-major bleed. In all analyses of risk of bleeding events, patients were censored at the time of starting permanent anticoagulation.

Age-specific (<75 years *vs* ≥75 years, and 5 year bands) risks of bleeding events were determined by site, severity, outcome (fatal, disabling, or non-disabling), and type of initial ischaemic event (cerebrovascular *vs* myocardial infarction), with further stratification by source of data (administrative coding alone *vs* all sources). Risks were presented both as an annual rate, which is an averaged rate (%) derived as number per 100 patient-years, or as cumulative risk (%).

We compared the risks of major bleeding in OXVASC patients after transient ischaemic attack or ischaemic stroke with the risks of major bleeding reported in trials of aspirin-based secondary prevention after transient ischaemic attack or ischaemic stroke individually and after pooling (Mantel–Haenszel–Peto method). Because the mean follow-up time in aspirin-based secondary prevention trials was 2·6 years, we used the 3 year risks in OXVASC.

We used Cox regression to determine predictors of major bleeding and of major upper gastrointestinal bleeding, adjusted for age, sex, and risk factors (appendix p 36). Risk factors that approached significance (p<0·10) in the age-adjusted and sex-adjusted regression were entered into a multivariable analysis. We also stratified the risks of major bleeding and major upper gastrointestinal bleeding by the externally derived Reduction of Atherothrombosis for Continued Health (REACH) bleeding score (appendix p 38)^20^ overall and by age (<75 years *vs* ≥75 years). Prognostic value was expressed as area under the receiver operator characteristic (ROC) curve.

In addition to risks of bleeding, we also determined the long-term risks of recurrent ischaemic vascular events (ischaemic stroke, myocardial infarction, and sudden cardiac death), with exclusion of patients with atrial fibrillation at baseline. We then compared the ratios of major bleeding risk with ischaemic event risk stratified by age and REACH score, and compared the 3 year ratios with reported ratios in aspirin-based secondary prevention trials. We also estimated the probable ratios of risk over benefits attributable to antiplatelet treatment in each age group (appendix p 36) on the basis of a previous systematic review.^5^ For upper gastrointestinal bleeding, we estimated the age-specific numbers needed to treat (NNT) with PPIs to prevent one bleed on the basis of the cumulative risks from the Kaplan–Meier curve,^21^ by use of the reported relative risk of 0·26 from a previous systematic review (appendix p 2).^6^ The appendix (p 36) provides details of sensitivity analyses. We did all analyses with SPSS (version 20).

### Role of the funding source

The funders of the study had no role in study design, data collection, data analysis, data interpretation, or writing of the report. The corresponding author had full access to all the data in the study and had final responsibility for the decision to submit for publication.

## Results

Of 3166 eligible patients, 1094 (35%) presented with myocardial infarction and 2072 (65%) presented with cerebrovascular events (table 1, appendix p 6). 1582 patients (50%) were aged 75 years or older and 577 (18%) were 85 years or older. Mortality follow-up was complete for all but six patients (<1%), and all but 29 patients (1%) had follow-up for non-fatal bleeding events.

**Table 1:** Baseline characteristics and 10 year risks of bleeding events requiring medical attention in patients given antiplatelet medication for secondary prevention

			**<75 years (n=1584)**	**≥75 years (n=1582)**	**Hazard ratio (95% CI)**	**p value**
Baseline characteristics*						
Age (years)			61·4 (10·0)	83·0 (5·4)	NA	<0·0001
Sex			..	..	..	<0·0001
	Male		1030 (65%)	687 (43%)	NA	..
	Female		554 (35%)	895 (57%)	NA	..
Type of index event			..	..	..	<0·0001
	Ischaemic stroke		511 (32%)	666 (42%)	NA	..
	Transient ischaemic attack		473 (30%)	422 (27%)	NA	..
	Non-ST-elevation myocardial infarction		333 (21%)	370 (23%)	NA	..
	ST-elevation myocardial infarction		267 (17%)	124 (8%)	NA	..
Premorbid use of antiplatelet treatment			462 (29%)	817 (52%)	NA	<0·0001
Premorbid gastric protection drugs†			323 (20%)	450 (28%)	NA	<0·0001
Post-event antiplatelet treatment			..	..	..	0·0137
	Aspirin-based		1529 (97%)	1498 (95%)	NA	..
	Non-aspirin-based		54 (3%)	82 (5%)	NA	..
**10 year risks of bleeding (n/patient-years)**						
All bleeds			179/7545	226/4509	1·76 (1·44–2·14)	<0·0001
	Severity					
		Significant non-major	122/7545	96/4509	1·11 (0·85–1·46)	0·44
		Major non-fatal	48/8050	95/4783	2·64 (1·86–3·74)	<0·0001
		Fatal	9/8249	35/5004	5·53 (2·65–11·54)	<0·0001
	Outcome (non-fatal bleeds)					
		Non-disabling	161/7545	139/4509	1·20 (0·96–1·51)	0·11
		Disabling	9/8215	52/4919	7·60 (3·74–15·47)	<0·0001
Upper gastrointestinal bleeds			52/7545	110/4509	2·94 (2·11–4·09)	<0·0001
	Severity					
		Significant non-major	28/7545	37/4509	1·88 (1·15–3·09)	0·0121
		Major non-fatal	21/8050	59/4783	3·76 (2·28–6·21)	<0·0001
		Fatal	3/8249	14/5004	6·67 (1·91–23·35)	0·003
	Outcome (non-fatal bleeds)					
		Non-disabling	46/7545	65/4509	1·97 (1·35–2·88)	0·0005
		Disabling	3/8215	31/4919	13·72 (4·18–45·02)	<0·0001
Intracranial bleeds			17/8172	28/4968	2·21 (1·21–4·05)	0·0102
Severity						
		Major non-fatal	13/8050	8/4783	0·79 (0·33–1·90)	0·60
		Fatal	4/8249	20/5004	7·14 (2·43–20·96)	0·0003
	Outcome (non-fatal bleeds)					
		Non-disabling	8/8172	2/4968	0·31 (0·07–1·47)	0·14
		Disabling	5/8219	6/4976	1·53 (0·47–5·00)	0·49
Other bleeds			110/7545	88/4509	1·12 (0·84–1·48)	0·44
	Severity					
		Significant non-major	94/7545	59/4509	0·88 (0·64–1·23)	0·46
		Major	16/8050	29/4783	2·46 (1·33–4·56)	0·0041
	Outcome					
		Non-disabling	107/7545	72/4509	0·94 (0·70–1·28)	0·71
		Disabling or fatal	3/8215	16/4919	7·11 (2·06–24·53)	0·0019

Data are mean (SD) or n (%), unless otherwise stated. Major bleeds were bleeds that were substantially disabling with persistent sequelae, intraocular bleeding leading to significant loss of vision, or bleeding requiring transfusion of 2 or more units of blood. NA=not applicable.

* The appendix (p 6) presents detailed baseline characteristics by age.

† Gastric protection drugs included proton-pump inhibitors or histamine_2_–receptor antagonist.

The 773 patients (24%) receiving premorbid gastric protection (PPI or histamine_2_-receptor antagonist; appendix p 7) were older than those not receiving protection and, after adjustment for age, were more likely to be anaemic and to have had previous peptic ulcer, vascular disease, hypertension, diabetes, and hyperlipidaemia (appendix p 7). Use of gastric protection increased to 32% (n=947) among 2914 survivors by 1-month follow-up and was maintained at 1-year follow-up (852 [33%] among 2583 survivors). At 1 year, 2301 patients (89%) were receiving antiplatelet treatment and 77 (3%) had switched to oral anticoagulants; at 5 years, 1042 (87%) of 1199 patients were receiving antiplatelet treatment and 19 (2%) were receiving anticoagulants.

Of 405 bleeding events (187 major bleeds) presenting to medical attention during 13 509 patient-years of follow-up, 162 (40%; 97 major bleeds) were upper gastrointestinal (appendix p 8). The average annual risk of bleeding was 3·36% (95% CI 3·04–3·70; 1·46%, 1·26–1·68 for major bleeds). Risks were similar after transient ischaemic attack or stroke versus myocardial infarction (appendix p 9), but the proportion of major bleeds that were intracerebral was higher (odds ratio [OR] 2·76, 95% CI 1·06–7·23; p=0·03). 390 bleeds (96%) occurred during antiplatelet treatment.

**Figure 1:**
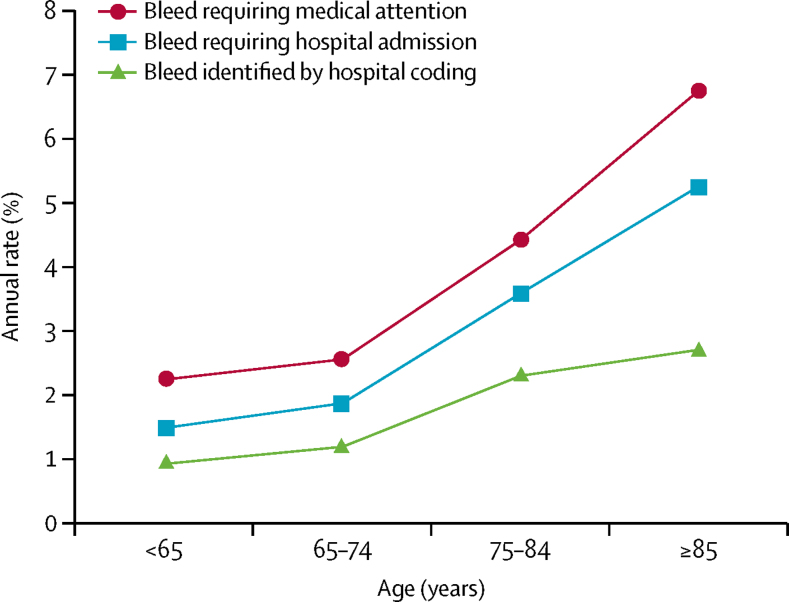
Annual rates of bleeding events requiring medical attention according to source of data Ascertainment in the Oxford Vascular Study, with multiple sources versus bleeding events identified by use of administrative hospital coding alone. Age-specific reasons for major bleeds that were not identified by administrative coding alone are reported in table 2.

Of the 314 bleeding events (78%) that required or occurred during hospital admission, 120 (38%) were not recorded in routine hospital administrative coding, whereas three (1%) non-bleeding events were miscoded as bleeds (figure 1, table 2). 73 (39%) of 187 major bleeding events were missed by routine coding alone (table 2), including 43 (44%) of 97 upper gastrointestinal bleeds, although 36 (80%) of 45 intracranial haemorrhages were identified.

**Table 2:** Age-specific reasons for major bleeds not identified by administrative coding alone

		**<65 years**	**65–74 years**	**75–84 years**	**≥85 years**	**Total**
**All bleeds**						
Bleeds requiring medical attention		95/856	84/728	149/1005	77/577	405/3116
Bleeds requiring (or during) hospital admission		65/95 (68%)	63/84 (75%)	124/149 (83%)	62/77 (81%)	314/405 (78%)*
Bleeds identified by administrative coding		41/95 (43%)	41/84 (49%)	82/149 (55%)	33/77 (43%)	197/405 (49%)
**Relevant coding details in major bleeds**						
Not identified by administrative coding		11/22 (50%)	13/35 (37%)	24/80 (30%)	25/50 (50%)	73/187 (39%)
	Codes for admission not found	5/11 (45%)	5/13 (38%)	10/24 (42%)	8/25 (32%)	28/73 (38%)
	In-hospital bleeds not coded but initial reason for admission coded	3/11 (27%)	4/13 (31%)	5/24 (21%)	13/25 (52%)	25/73 (34%)
	Coding found for another bleed only	0	0	2/24 (8%)	0	2/73 (3%)
	Coding for related diagnosis only†	3/11 (27%)	4/13 (31%)	7/24 (29%)	4/25 (16%)	18/73 (25%)
Identified by administrative coding		11/22 (50%)	22/35 (63%)	56/80 (70%)	25/50 (50%)	114/187 (61%)

See figure 1. Data are n/N or n/N (%). Major bleeds were bleeds that were substantially disabling with persistent sequelae, intraocular bleeding leading to significant loss of vision, or bleeding requiring transfusion of 2 or more units of blood.

* 91 patients that required medical attention were not admitted but presented to general practitioners (n=71) or treated in hospital without being admitted overnight (n=20).

† Related diagnosis included anaemia, diverticular disease, oesophagitis, or gastritis.

**Figure 2:**
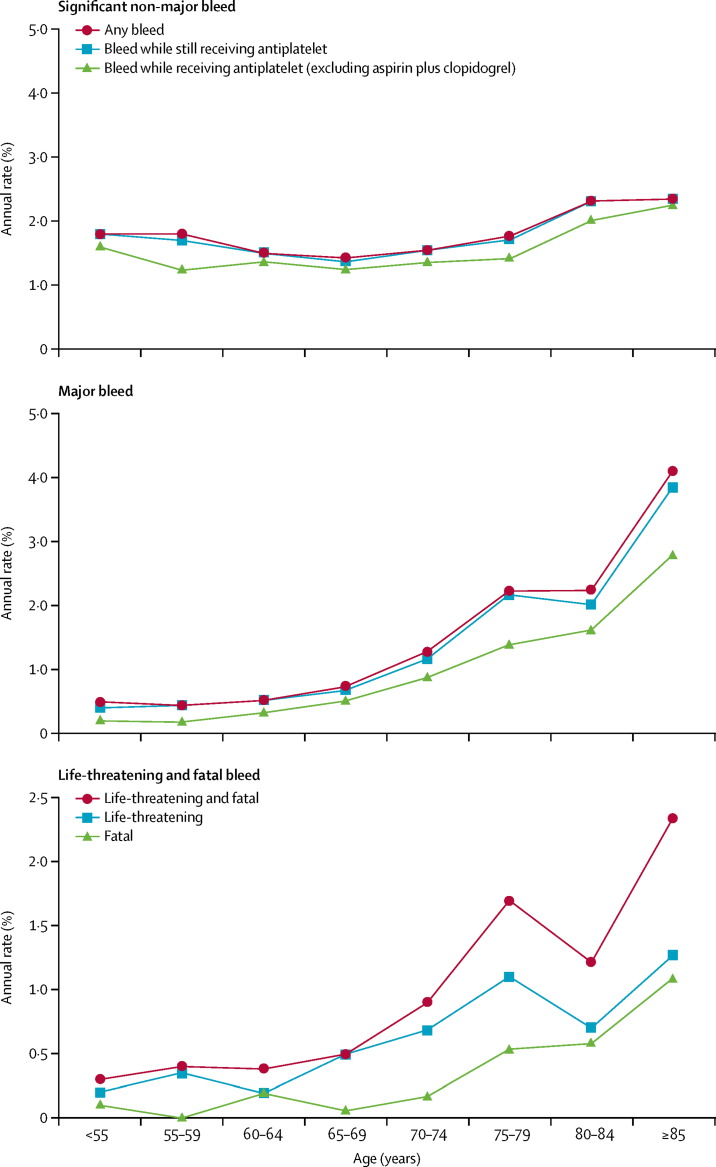
Age-specific annual rate of bleeding events requiring medical attention Stratified by severity and by antiplatelet treatment immediately before the event. Annual rate derived as number per 100 patient-years. We used Clopidogrel in Unstable angina to prevent Recurrent Events (CURE) criteria to define bleeding events as major (substantially disabling with persistent sequelae, intraocular bleeding leading to significant loss of vision, or bleeding requiring transfusion of ≥2 units of blood) and life-threatening or fatal (symptomatic intracranial haemorrhage, fall in haemoglobin of ≥5 g/dL, hypotension requiring intravenous inotropes, or required surgical intervention or transfusion of ≥4 units of blood).

Risk of non-major bleeds was unrelated to age and risk of major bleeds did not increase with age in patients younger than 70 years (figure 2, appendix p 10). The mean age of patients in previous trials of aspirin for secondary prevention of transient ischaemic attack or stroke was 63 years, and most were younger than 75 years (appendix pp 3–5, 11). The average annual risk of major bleeding in OXVASC patients with transient ischaemic attack or stroke younger than 75 years during the first 3 years of follow-up (1·1%, 95% CI 0·7–1·6; mean age 62 years [SD 10·3]) was similar to the annual risk of major bleeding reported in the previous trials (pooled risk 1·0%, 0·8–1·1; appendix pp 11, 12).

The annual risk of major bleeds in OXVASC increased steeply above age 70 years (figure 2), reaching 4·1% at age 85 years or older, with similar patterns for both life-threatening and fatal bleeds, reflecting high risks of upper gastrointestinal and intracranial bleeds at older ages (appendix p 10). Results were similar in analyses confined to patients still receiving antiplatelet treatment and in analyses excluding bleeds occurring during treatment with aspirin plus clopidogrel (figure 2, appendix p 13). The antiplatelet regimen at the time of bleeding did not differ with age (appendix p 14).

The annual risk of major bleeding was higher in patients aged 75 years or older at baseline than in those younger than 75 years, both at 3 years (hazard ratio [HR] 2·73, 1·95–3·82; p<0·0001) and at 10 years (3·10, 2·27–4·24; p<0·0001). The increased 10 year risk was most prominent for upper gastrointestinal bleeds (4·13, 2·60–6·57; p<0·0001; appendix p 15). Results were similar between patients with transient ischaemic attack or stroke and those with myocardial infarction (appendix p 16).

**Figure 3:**
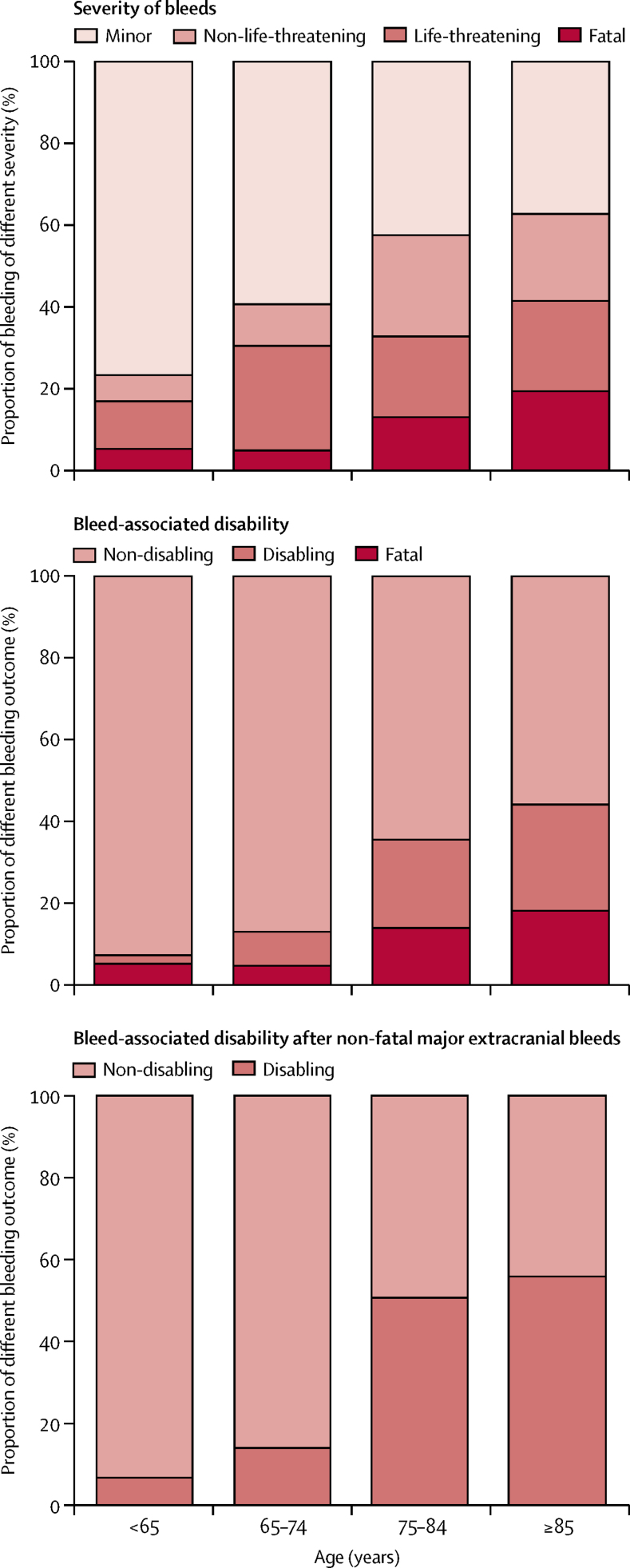
Distributions by age of severity of bleeding events requiring medical attention and of new or worsening disability attributable to bleeds

Patients aged 75 years or older also had more severe bleeds than those aged younger than 75 years (p_trend_<0·0001; table 1, figure 3).

The outcome of non-fatal bleeds was also worse at older ages (table 1, figure 3, appendix pp 17, 18 lists the reasons for bleed-associated disability or death). The proportion of survivors in whom an extracranial bleed resulted in new or a sustained increase in disability increased with age (four [3%] of 157 patients <75 years *vs* 46 [25%] of 183 patients ≥75 years; OR 12·8, 95% CI 4·5–36·6; p<0·0001), particularly after major upper gastrointestinal bleeds (three [14%] of 21 *vs* 31 [53%] of 59 patients; disability or death: six [25%] of 24 *vs* 45 [62%] of 73 patients; table 1), such that the long-term risk of disabling or fatal upper gastrointestinal bleeding was ten times higher at age 75 years or older (HR 10·26, 95% CI 4·37–24·13, p<0·0001; absolute risk 9·15, 95% CI 6·67–12·24 per 1000 patient-years), substantially outnumbering disabling or fatal intracerebral bleeds (n=18).

The annual risk of bleeding events reduced over time (appendix p 9), both in patients treated initially with aspirin plus clopidogrel and in those patients on monotherapy (appendix p 19), after both myocardial infarction and cerebrovascular events (appendix p 9), and after exclusion of the first 90 days after index events (appendix p 20). However, the risks of major bleeding were more sustained in patients aged 75 years or older than in those younger than 75 years (time-dependent interaction p=0·032; appendix p 21), even in those receiving premorbid antiplatelet drugs already at baseline (appendix p 22) and for major upper gastrointestinal bleed (appendix p 23). The risk was most sustained in patients aged 75–84 years (appendix p 22).

The associations of major bleeding and major upper gastrointestinal bleeding with age were independent of sex, history of vascular disease, vascular risk factors, and history of peptic ulcer (table 3). The 5 year risk increased with the REACH risk score in patients younger than 75 years (appendix p 24; major bleeds area under the ROC curve 0·61, 95% CI 0·54–0·69; p=0·004; major upper gastrointestinal bleeds 0·65, 0·54–0·76; p=0·014), but not at age 75 years or older (major bleed 0·51, 0·46–0·56; p=0·69; major upper gastrointestinal bleed 0·50, 0·43–0·57; p=0·93).

**Table 3:** Univariate and multivariate analyses for risk factors for major bleeding events and for major upper gastrointestinal bleeding

	**Major bleed**						**Major upper gastrointestinal bleed**					
	Univariate analysis		Adjusted for age and sex		Multivariate analysis*		Univariate analysis		Adjusted for age and sex		Multivariate analysis*	
	HR (95% CI)	p value	HR (95% CI)	p value	HR (95% CI)	p value	HR (95% CI)	p value	HR (95% CI)	p value	HR (95% CI)	p value
Age (per 10 years)	1·68 (1·47–1·93)	<0·0001	1·72 (1·50–1·97)	<0·0001	1·72 (1·47–2·02)	<0·0001	1·82 (1·50–2·22)	<0·0001	1·86 (1·53–2·26)	<0·0001	1·97 (1·59–2·45)	<0·0001
Female	1·14 (0·86–1·52)	0·37	0·84 (0·62–1·13)	0·24	0·90 (0·67–1·22)	0·50	1·18 (0·79–1·76)	0·42	0·83 (0·55–1·25)	0·38	0·91 (0·60–1·37)	0·64
Weight (per kg)	0·98 (0·97–0·99)	0·0006	0·99 (0·98–1·00)	0·17	..	..	0·98 (0·97–1·00)	0·0164	1·00 (0·98–1·01)	0·53	..	..
History of vascular disease†	1·69 (1·25–2·27)	0·0006	1·33 (0·98–1·80)	0·06	1·16 (0·85–1·57)	0·36	1·93 (1·29–2·90)	0·0015	1·50 (0·99–2·25)	0·05	1·23 (0·81–1·87)	0·33
Hypertension	1·52 (1·13–2·05)	0·0056	1·28 (0·95–1·73)	0·10	..	..	1·50 (1·00–2·27)	0·05	1·25 (0·83–1·89)	0·29	..	..
Diabetes	1·60 (1·11–2·29)	0·0108	1·68 (1·17–2·41)	0·0047	1·46 (1·01–2·13)	0·05	1·67 (1·02–2·74)	0·0401	1·79 (1·09–2·93)	0·0210	1·46 (0·87–2·45)	0·15
Hyperlipidaemia	1·11 (0·82–1·50)	0·52	1·14 (0·84–1·54)	0·41	..	..	1·12 (0·74–1·72)	0·59	1·17 (0·77–1·79)	0·47	..	..
Current smoking‡	0·86 (0·59–1·25)	0·42	1·54 (1·03–2·30)	0·0370	1·61 (1·07–2·42)	0·0224	1·15 (0·71–1·86)	0·58	2·32 (1·38–3·89)	0·0014	2·39 (1·41–4·02)	0·001
Alcohol >14 units per week§	0·88 (0·58–1·33)	0·55	1·22 (0·79–1·88)	0·37	..	..	1·02 (0·59–1·77)	0·95	1·51 (0·85–2·70)	0·16	..	..
Anaemia¶	1·74 (1·26–2·39)	0·0007	1·53 (1·11–2·11)	0·0095	1·31 (0·94–1·83)	0·11	2·25 (1·48–3·43)	0·0002	1·96 (1·29–3·00)	0·0018	1·58 (1·02–2·45)	0·0429
History of cancer	2·22 (1·55–3·18)	<0·0001	1·75 (1·22–2·52)	0·0026	1·86 (1·29–2·69)	0·0009	2·48 (1·52–4·02)	0·0002	1·91 (1·17–3·11)	0·0098	2·06 (1·25–3·38)	0·0043
Chronic liver disease	2·23 (0·92–5·43)	0·08	2·81 (1·15–6·84)	0·0232	2·35 (0·95–5·77)	0·06	3·47 (1·27–9·43)	0·0150	4·53 (1·66–12·37)	0·0032	3·77 (1·37–10·42)	0·0104
Renal failure‖	5·80 (2·96–11·36)	<0·0001	5·54 (2·83–10·85)	<0·0001	4·69 (2·32–9·48)	<0·0001	8·87 (4·10–19·21)	<0·0001	8·49 (3·92–18·38)	<0·0001	7·20 (3·17–16·39)	<0·0001
Atrial fibrillation	2·09 (1·41–3·09)	0·0002	1·38 (0·93–2·07)	0·11	..	..	1·82 (1·03–3·21)	0·0393	1·14 (0·64–2·04)	0·66	..	..
Chronic heart failure	2·08 (1·37–3·15)	0·0006	1·44 (0·94–2·19)	0·09	1·23 (0·80–1·89)	0·35	1·79 (0·98–3·28)	0·06	1·18 (0·64–2·19)	0·59	..	..
History of peptic ulcer	1·99 (1·32–2·99)	0·0010	1·67 (1·11–2·52)	0·0140	1·61 (1·07–2·44)	0·0244	2·32 (1·36–3·96)	0·0021	1·93 (1·13–3·32)	0·0167	1·79 (1·04–3·10)	0·0369
Premorbid antiplatelet	1·47 (1·10–1·96)	0·0090	1·09 (0·81–1·46)	0·57	..	..	1·49 (1·00–2·22)	0·05	1·07 (0·72–1·61)	0·73	..	..
Dual antiplatelet post-event**	0·82 (0·61–1·10)	0·18	0·96 (0·71–1·30)	0·81	..	..	0·77 (0·51–1·17)	0·22	0·93 (0·61–1·43)	0·75	..	..
Premorbid PPI/H2-antagonist	1·35 (0·99–1·86)	0·06	1·15 (0·84–1·59)	0·38	..	..	1·16 (0·73–1·82)	0·53	0·97 (0·62–1·53)	0·90	..	..

Major bleeds were bleeds that were substantially disabling with persistent sequelae, intraocular bleeding leading to significant loss of vision, or bleeding requiring transfusion of 2 or more units of blood. HR=hazard ratio. PPI=proton-pump inhibitor. H2-antagonist=histamine_2_–receptor antagonist.

* Risk factors that approached significance (p<0·10) in the age-adjusted and sex-adjusted regression were entered into multivariable Cox regression analysis.

† History of stroke, transient ischaemic attack, myocardial infarction, or peripheral vascular disease.

‡ Data missing for 21 patients.

§ Data missing for 202 patients.

¶ Baseline haemoglobin less than 13 g/L in men and 12 g/L in women.

‖ Glomerular filtration rate of less than 30 mL/min, estimated with the Cockroft and Gault formula.

** Mainly aspirin and clopidogrel, and was routinely prescribed for a short period after the index event.

489 non-fatal and 208 fatal ischaemic vascular events occurred during follow-up. The absolute risks of major bleeding versus ischaemic events increased with age and REACH score (appendix p 25). In patients younger than 75 years, the ratio of major bleeds to ischaemic events (0·20, 95% CI 0·14–0·27) was similar to the ratios in previous aspirin trials (pooled ratio 0·19, 0·17–0·21; appendix p 11). However, the ratio in OXVASC increased with age (75–84 years 0·32, 0·23–0·43; ≥85 years 0·46, 0·32–0·67; appendix pp 25, 26), and the risk of major bleeds estimated to be attributable to antiplatelet treatment approached the risk of ischaemic events estimated to have been prevented (appendix pp 27). Results were consistent in sensitivity analysis censoring at the time of either a first major bleed or a first ischaemic event (appendix pp 28, 29). Moreover, severity of recurrent ischaemic strokes did not increase with age, with less than half (101 [47%] of 213) being disabling or fatal at age 75 years or older.

In the only published meta-analysis^6^ of randomised trials of PPIs versus placebo in patients taking antiplatelet drugs (predominantly aspirin), PPI use reduced upper gastrointestinal bleeding by 74% (appendix p 2). When we used this estimate, the NNT with PPIs to prevent one major upper gastrointestinal bleed at 5 year follow-up decreased with increasing age: 80 for patients younger than 65 years, 75 for patients aged 65–74 years, 23 for patients aged 75–84 years, and 21 for patients aged 85 years or older (appendix p 30). The NNT with PPIs to prevent one disabling or fatal upper gastrointestinal bleed at 5 year follow-up also decreased, from 338 for patients younger than 65 years to 25 for patients aged 85 years or older (appendix p 30). The outcomes were similar in analyses excluding patients with history of peptic ulcer or those receiving premorbid gastric protection treatment (appendix p 31).

## Discussion

In this large prospective population-based study, the long-term risks and severity of bleeding in patients receiving predominantly aspirin-based secondary prevention increased steeply with age. Although the risks of major bleeding in patients aged younger than 75 years were similar to the risks in previous trials of aspirin and other antiplatelet drugs, the risks at older ages were higher and more sustained than at younger ages, and the functional outcome was much worse, with a substantial risk of disabling or fatal upper gastrointestinal bleeding.

The increase with age in risk of bleeding on antiplatelet treatment is in line with previous studies.^13^, ^14^, ^15^, ^22^ One retrospective cohort study^23^ showed that antithrombotic-associated gastrointestinal bleeds had high rates of hospital admission and transfusion at older ages, one study^15^ found that in patients aged 85 years or older the gastrointestinal tract was the most common location of fatal haemorrhage after ischaemic stroke, and one study^24^ reported higher rates of complications and peptic ulcer-related mortality in elderly patients, but these studies did not report age-specific data for functional outcome or case fatality from upper gastrointestinal bleeding. We have shown that severity, case fatality, and poor functional outcome increase steeply with age.

The mechanisms underlying poor outcomes after upper gastrointestinal bleeding at older ages are multifactorial.^25^ We found that complications, such as thrombotic events and worsening heart failure, were common, but the reasons for functional decline were often unclear. Further research is needed to understand the determinants of poor outcomes, including the effect of withdrawal versus continuation of antiplatelet treatment in the acute phase,^26^, ^27^, ^28^ but our results highlight the need for more rigorous prevention of bleeds in the first place.

In relation to prevention of upper gastrointestinal bleeding, research into the effectiveness of *Helicobacter pylori* screening and eradication in older patients already receiving antiplatelet treatment is underway,^29^ but gastric protection strategies otherwise focus on PPIs, which reduce upper gastrointestinal bleeding by 70–90% in patients receiving long-term antiplatelet treatment.^6^, ^30^ However, consistent with other UK or European studies,^9^, ^10^ PPI use in patients receiving long-term antiplatelet treatment in our study was only about 30%. We did not routinely co-prescribe PPIs, partly because clinical guidelines on secondary prevention of vascular events make no specific recommendations on PPI use,^3^, ^4^ and partly because no accepted definition exists of patients at high risk of upper gastrointestinal bleeding.^12^ However, our results suggest that in secondary prevention setting, an age of 75 years or older alone is sufficient to define patients at high-risk, with a reasonable NNT to prevent one major upper gastrointestinal bleed. Age 75 years would be an appropriate threshold to start a PPI both in patients newly initiated on antiplatelet drugs and in patients on established treatment. Use of an age-based criterion is also supported by the poor performance of the REACH score in further risk stratification at older ages, although our risk factor analysis suggests that prognostication could be improved. Moreover, we limited our estimation of the potential benefits of routine co-prescription of PPIs only to acute bleeding events, but anaemia and peptic ulcer perforation without gastrointestinal bleeding should also be reduced.^6^

Non-upper gastrointestinal bleeds constituted 60% of all bleeds and 48% of major bleeds in our study population, highlighting the importance of prevention, particularly control of blood pressure in preventing intracranial bleed.

As expected, the risk of recurrent ischaemic events also increased with age, but the relative excess of ischaemic over major bleeding events diminished. Moreover, in patients aged 75 years or older, major upper gastrointestinal bleeding was at least as likely to be disabling or fatal as recurrent ischaemic stroke. In secondary prevention of vascular disease, aspirin reduces the long-term relative risk of major ischaemic events by about 20%, but doubles the relative risk of major bleeding.^5^ In our study, for patients younger than 75 years, the ratios of major bleeding over ischaemic risk were similar to those in previous randomised trials of aspirin. However, this ratio increased with age, raising questions about the balance of risk and benefit of long-term antiplatelet treatment in this age group if a PPI is not co-prescribed. Although short-term benefit from antiplatelet treatment is clear,^31^ and sudden discontinuation is hazardous,^26^, ^27^, ^28^ a trial of gradual withdrawal would be justifiable in patients for whom long-term gastric protection is not acceptable.

Strengths of our study are its prospective population-based design with inclusion of all treated patients irrespective of age and frailty, long-term face-to-face follow-up, reliable ascertainment of bleeding events through multiple sources, and assessment of functional outcome, but there are some limitations. First, some analyses combined patients with cerebrovascular and coronary vascular events, although the early risk of bleeding might be higher after coronary events because of interventional strategies and greater use of dual antiplatelet therapy. However, the risk of bleeding and the association with age were similar when only bleeds occurring with single antiplatelet treatment were included. Second, although we had data for all prescribed medication of follow-up, and very few bleeds occurred secondary to prescribed non-steroidal anti-inflammatory drugs (NSAIDs) or corticosteroids, we might have missed data for over-the-counter use of NSAIDs. Third, we found that non-major significant bleeding was unrelated to age, but older patients might be less likely to identify and report minor bleeding. However, any under-ascertainment of more minor bleeds at older ages would not alter our conclusions about the absolute risk of major bleeding. Fourth, we defined severity of bleeds using the CURE trial criteria, but no consensus exists about the optimal categorisation.^32^ Fifth, the estimated age-specific NNT for PPI use in prevention of upper gastrointestinal bleeds was based on the assumptions that the efficacy of PPIs was the same for prevention of any bleed versus major bleed, was similar at different ages, and remained consistent with time. Sixth, our results focused mainly on aspirin-based antiplatelet treatment in secondary prevention because only a few of our patients were receiving long-term clopidogrel. However, the only previous large randomised trial^33^ of aspirin versus clopidogrel in secondary prevention of vascular events showed no significant difference in risk of major bleeding. Seventh, the predominant aspirin formulation in our cohort was 75 mg enteric coated, which will limit generalisability to countries in which other doses or formulations are common. Eighth, we did not attempt to estimate the number needed to harm in relation to potential adverse effects of long-term PPI use. In the absence of large randomised trials of long-term treatment, reliable estimation of any hazard is difficult (appendix p 39 lists detailed discussion for harms).^7^, ^34^, ^35^, ^36^ Ninth, for the estimated net benefit of antiplatelet treatment, our estimates might have been conservative. We applied the reported two-times increased risk of major bleeding on aspirin in secondary prevention trials to all age groups and to all types of bleeding in our non-trial population. Finally, in our multivariable analyses of the association of age and major bleeding, we adjusted for known risk factors, but residual confounding cannot be excluded.

In conclusion, in secondary prevention with aspirin-based antiplatelet treatment without routine PPI use, the long-term risk of bleeding at age 75 years or older is higher and more sustained than in the younger age groups included in previous trials, with particularly high risks of disabling or fatal upper gastrointestinal bleeding. Given that half of the major bleeds in patients aged 75 years or older were upper gastrointestinal, the estimated NNT for routine PPI use to prevent major upper gastrointestinal bleed is low and co-prescription should be considered in future secondary prevention guidelines. More research is still required into how best to identify patients at high risk of bleeding, how to reduce the risk of non-upper gastrointestinal bleeds, and into the overall balance of risks and benefits of long-term antiplatelet treatment at older ages in both primary and secondary prevention.
