# Supplementary material for: Age-specific risks, severity, time course, and outcome of bleeding on long-term antiplatelet treatment after vascular events: a population-based cohort study
Source: Lancet. 2017 Jul 29;390(10093):490–9. doi: 10.1016/S0140-6736(17)30770-5 (PMC5537194; doi:10.1016/S0140-6736(17)30770-5)
Supplement: Supplementary appendix [file mmc1.pdf]

THE LANCET

## Supplementary appendix

This appendix formed part of the original submission and has been peer reviewed. We post it as supplied by the authors.

Supplement to: Li L, Geraghty OC, Mehta Z, Rothwell PM, on behalf of the Oxford Vascular Study. Age-specific risks, severity, time course, and outcome of bleeding on long-term antiplatelet treatment after vascular events: a population-based cohort study. *Lancet* 2017; published online June 13. http://dx.doi.org/10.1016/S0140-6736(17)30770-5.

# Age-specific risks, severity, time-course and outcome of bleeding on long-term antiplatelet treatment after vascular events: population-based cohort study

Linxin Li†, Olivia C Geraghty†, Ziyah Mehta, Peter M Rothwell, on behalf of the Oxford Vascular Study

†Both authors contributed equally

Centre for Prevention of Stroke and Dementia, Nuffield Department of Clinical Neurosciences, John Radcliffe Hospital, University of Oxford, UK

**Correspondence:**

Prof. Peter M. Rothwell
Centre for Prevention of Stroke and Dementia
Nuffield Department of Clinical Neurosciences
Level 6, West Wing
John Radcliffe Hospital
Headley Way, Headington
Oxford
OX3 9DU
Tel. +44 (0)1865 231610
E-mail Peter.rothwell@ndcn.ox.ac.uk

## Web appendix

**Age-specific risks, severity, time-course and outcome of bleeding on long-term antiplatelet treatment after vascular events: population-based study**


| Web appendix | Title | Page |
|---|---|---|
| 1 | Summary of PPI trials in the prevention of antiplatelet-associated upper-GI complications | 2 |
| 2a-c | Comparison of the age and follow-up lengths of patients in aspirin trials (aspirin vs. placebo) for secondary prevention vs. OXVASC | 3-5 |
| 3 | Baseline characteristics of patients with myocardial infarction,TIA and ischaemic stroke in OXVASC who were initially treated with antiplatelet medication for secondary prevention stratified by age | 6 |
| 4 | Baseline charateristics of patients on premorbid gastric protection vs. those not on gastric protection | 7 |
| 5 | Sites of bleeding events requieing medical attention stratified by severity of bleeds (CURE criteria) | 8 |
| 6 | Long-term risks of bleeding events requiring medical attention stratified by severity, site of bleeding and index event type | 9 |
| 7 | Age-specific risks of bleeding events requiring medical attention stratified by sites and severity of bleeding | 10 |
| 8 | Risks of major bleeding vs. recurrent ischaemic vascular events in Aspirin-controlled trials for secondary prevention of stroke | 11 |
| 9 | Annual rates of major bleeding in Aspirin-controlled trials for secondary prevention of stroke vs. OXVASC (cerebrovascular cohort) | 12 |
| 10 | Annual rates of life-threatening and fatal bleeding stratified by age (left panels) and the distributions of bleeding of different severity by age (right panels) | 13 |
| 11 | Types of the antiplatelet agents patients were taking immediately prior to the bleeding events that required medical attention stratified by age | 14 |
| 12 | Risks of major bleeding in patients ≥75 years vs. patients <75 years stratified by site of bleeding | 15 |
| 13 | Risks of major bleeding in patients ≥75 years vs. patients <75 years stratified by type of the index event | 16 |
| 14 | Reasons for new or worsening disability attributable to the bleeding events | 17 |
| 15 | Reasons for death attributable to the bleeding events | 18 |
| 16 | Long-term risks of bleeding requiring medical attention stratified by the number of the initial antiplatelet treatment | 19 |
| 17 | Sensitivy analyses of the long-term risks of bleeding events requiring medical attention stratified by severity, site of bleeding and the index event type excluding the first 90 days | 20 |
| 18 | Time-course of risks of major bleeding events in patients ≥75 years versus patients <75 years | 21 |
| 19 | Age-specific time-course of major bleeding stratified by premorbid antiplatelet drug use at baseline (A) and in patients who had been stable on antiplatelet treatment for one year (B) | 22 |
| 20 | Time-course of major bleeding in patients aged ≥ 75 years vs. patients aged <75 years stratified by site of bleeding | 23 |
| 21 | Cumulative 5-year risk of major bleeds and major upper-GI bleeds in OXVASC stratified by the REACH bleeding risk score and by age | 24 |
| 22 | Observed 5-year risks of major bleeding versus recurrent ischaemic events stratified by age and by the REACH bleeding risk score | 25 |
| 23 | Observed age-specific 5-year risks of major bleeding vs. ischaemic events in OXVASC stratified by type of the index event | 26 |
| 24 | Estimated 5-year risk of major bleeding attributable to antiplatelet treatment vs. ischaemic events prevented by antiplatelet treatment stratified by age | 27 |
| 25 | Sensitivity analyses censoring at a composite outcome of major bleeding events or recurrent ischaemic events for the observed age-specific 5-year risks of major bleeding vs. ischaemic events | 28 |
| 26 | Sensitivity analyses censoring at a composite outcome of major bleeding events or recurrent ischaemic events for the estimated age-specific 5-year risk of bleeding attributable to antiplatelet treatment vs. ischaemic events prevented by antiplatelet treatment | 29 |
| 27 | Estimated age-specific numbers-needed-to-treat (NNT) to prevent one upper-GI bleed based on different lengths of follow-up in patients on antiplatelet treatment for secondary prevention of vascular events | 30 |
| 28 | Sensitity analyses of the estimated age-specific numbers-needed-to-treat (NNT) to prevent one upper-GI bleed based on different lengths of follow-up in patients on antiplatelet treatment for secondary prevention of vascular events excluding patients with previous peptic ulcer or excluding patients on premorbid gastric protection agents | 31 |
| 29 | References for PPI trials in the prevention of antiplatelet-associated upper-GI complications | 32 |
| 30 | References for aspirin-controlled secondary prevention trials | 33 |
| 31 | Additional Methods on OXVASC methodology, statistical analyses, and sensitivity analyses | 34 |
| 32 | Additional Methods on criteria and risk scores used in the study | 37 |
| 33 | Additional Discussion on potential harms of PPI | 39 |

**Web appendix 1. Summary of PPI trials in the prevention of antiplatelet-associated upper-GI complications.**

| Study | Year | Country | Antiplatelet treatment | Primary/secondary | Patient characteristics | Mean age |
|---|---|---|---|---|---|---|
| **vs. Placebo** | | | | | | |
| Lai[e1] | 2002 | Hong Kong | low-dose Aspirin | Secondary | High-risk/endoscopic proved history of peptic ulcer | 70 |
| AstraZeneca [e2] | NR | Japan | low-dose Aspirin | NR | High-risk/history of peptic ulcer | NR |
| Scheiman [e3] | 2011 | 20 countries | low-dose Aspirin | Both | High-risk/history of peptic ulcer or age >65yrs or age >60yrs with GI symptoms and erosions or aspirin naïve patients | 67 |
| Hsu [e4] | 2011 | Taiwan | Clopidogrel | Secondary | High-risk/history of peptic ulcer but normal endoscopic exam at entry/cannot take long-term aspirin | 72 |
| Yeomans [e5] | 2008 | 10 countries | low-dose Aspirin | Secondary | Non-high risk/older than 60yrs/no baseline peptic ulcer on endoscopy | 69 |
| Bhatt [e6] | 2010 | 15 countries | A+C | Secondary | Non-high risk | 68 |
| **vs. other gastric protection** | | | | | | |
| Ng [e7] | 2010 | Hong Kong | low-dose Aspirin | Secondary | High-risk/history of peptic ulcers or erosions with or without bleeding | 69 |
| Sugano [e8] | 2011 | Japan | low-dose Aspirin | Secondary | High-risk/endoscopic proved history of peptic ulcer | 69 |
| Sanuki [e9] | 2012 | Japan | low-dose Aspirin | Secondary | High-risk/endoscopic proved history of peptic ulcer | 73 |
| Chan [e10] | 2001 | Hong Kong | low-dose Aspirin | Secondary | High-risk/endoscopic proved acute peptic ulcer | 69 |
| Ng [e11] | 2012 | Hong Kong | A+C+others | Secondary | Non-high risk | 63 |

| Study | Treatment | Control | Primary endpoint | Follow-up | Incidence/treatment | Incidence/control | Hazard Ratio (95%CI)† |
|---|---|---|---|---|---|---|---|
| **vs. Placebo** | | | | | | | |
| Lai[e1] | Lansoprazole | Placebo | Clinical/ulcer complications | 1 year | 1.6 | 14.8 | 10.6(1.3-86.1)* |
| AstraZeneca [e2] | Esomeprazole | Placebo | Peptic ulcer | 1 year | 1.7 | 18.8 | 0.09 (0.02-0.41) |
| Scheiman [e3] | Esomeprazole 20mg/40mg | Placebo | Endoscopic/gastric or duodenal ulcer | 26 weeks | 1.1/1.5 | 7.4 | 0.14 (0.07-0.30)/0.19 (0.10-0.37) |
| HSU [e4] | Esomeprazole | Placebo | Endoscopic/gastric or duodenal ulcer | 6 months | 1.2 | 11 | NR |
| Yeomans [e5] | Esomeprazole | Placebo | Endoscopic/gastric or duodenal ulcer | 26 weeks | 1.8 | 6.2 | NR |
| Bhatt [e6] | Omeprazole | Placebo | Clinical/composite of upper-GI events | 180 days | 1.1 | 2.9 | 0.34 (0.18-0.63) |
| **vs. other gastric protection** | | | | | | | |
| Ng [e7] | Pantoprazole | Famotidine | Clinical/ulcer complications | 48 weeks | 0 | 20 | NR |
| Sugano [e8] | Lansoprazole | Gefarnate | Endoscopic/gastric or duodenal ulcer | 7.5m/5.7m (median) | 3.7 | 31.7 | 0.10 (0.04-0.23) |
| Sanuki [e9] | Rabeprazole 10mg/20mg | Gefarnate | Endoscopic/gastric or duodenal ulcer | 12 weeks | 5.5 | 26.7 | 0.19 (0.08-0.39) |
| Chan [e10] | Omeprazole | HP eradication+placebo | Clinical or Endoscopic /GI bleed or Hb drop of 2g/dl | 6 months | 0.9 | 1.9 | NR |
| Ng [e11] | Esoprazole | Famotidine | Clinical/upper-GI bleed, perforation or obstruction | 19.2 /17.6 weeks | 0.6 | 6.1 | 0.10 (0.01-0.50) |

PPI=proton pump inhibitor, A=aspirin, C=Clopidogrel, GI=gastrointestinal, Hb=haemoglobin, NR=not reported, HP=H.pylori. *reported HR for control vs. treatment. †pooled relative risk=0.26 from a systematic review (reference 6 in the main paper).

**Web appendix 2a. Comparison of the age and follow-up lengths of patients in aspirin trials (aspirin vs. placebo) for secondary prevention vs. OXVASC.**

| Studies | Publication year | Number of participants | Follow-up (median) | Mean age | Age range | ≥75 years% |
|---|---|---|---|---|---|---|
| **Post-MI** | | | | | | |
| Secondary prevention trials | | | | | | |
| Cardiff-I[e26] | 1974 | 1239 | 2 years | 55 | NR | NR but ≥65 years: 168 (13.6) |
| Cardiff-II[e27] | 1979 | 1725 | 1 years | 56 | NR | 27 (1.6) |
| PARIS-I[e28] | 1980 | 1216 | 41 months | 56 | NR | NR but ≥55 years: 694 (57.1) |
| AMIS[e29] | 1980 | 4524 | 38 months | 55 | 30-69 | 0 |
| CDP-A[e30] | 1976 | 1529 | 22 months | 56 | 30-64 | 0 |
| Gamis[e31] | 1980 | 626 | 2 years | 59 | 45-70 | 0 |
| OXVASC-MI cohort | | 1094 | 3.6 years | 71 | 29-101 | 494 (45.2) |
| **Post-TIA/stroke** | | | | | | |
| Secondary prevention trials | | | | | | |
| AITIA[e12,e25] | 1975 | 319 | 1 years | 58 | NR | 22 (6.9) |
| UK-TIA[e13] | 1988 | 2435 | 4 years | 60 | ≥40 | NR |
| Reuther[e32] | 1976 | 60 | 2 years | 58 | NR | NR |
| CA Co-op[e33] | 1978 | 283 | 3 years | 61 | NR | NR |
| Toulouse TIA[e34] | 1982 | 303 | 3 years | 63 | NR | NR |
| AICLA[e24] | 1981 | 402 | 3 years | 64 | NR | 64 (15.9) |
| Danish Co-op[e35] | 1980 | 203 | 25 months | 59 | 34-75 | 0 |
| Swedish Co-op[e16] | 1987 | 505 | 2 years | 68 | 27-93 | NR but >65: 243 (48.1) |
| Danish low dose[e36] | 1986 | 301 | 23 months | 59 | NR | NR |
| SALT[e15] | 1991 | 1363 | 32 months | 67 | 50-79 | NR |
| ESPS-2[e17] | 1996 | 3298 | 2 years | 67 | ≥18 | 895 (27.1) |
| OXVASC-TIA/stroke cohort | | 2072 | 3.7 years | 73 | 21-99 | 1088 (52.5) |

NR= not reported. MI=myocardial infarction.

**Web appendix 2b. Age of patients in aspirin trials (aspirin vs. placebo) for secondary prevention vs. OXVASC.** (Trials plotted by year of recruitment and the size of the circle represents the size of the study; MI=myocardial infarction; CVA=cerebrovascular disease)

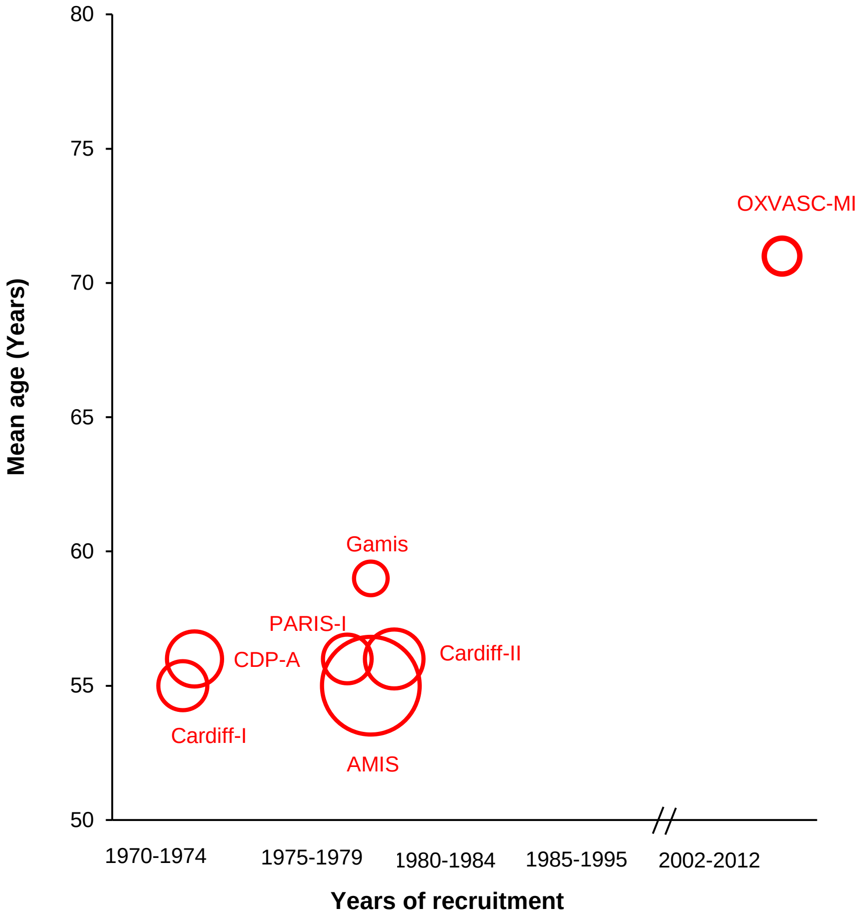


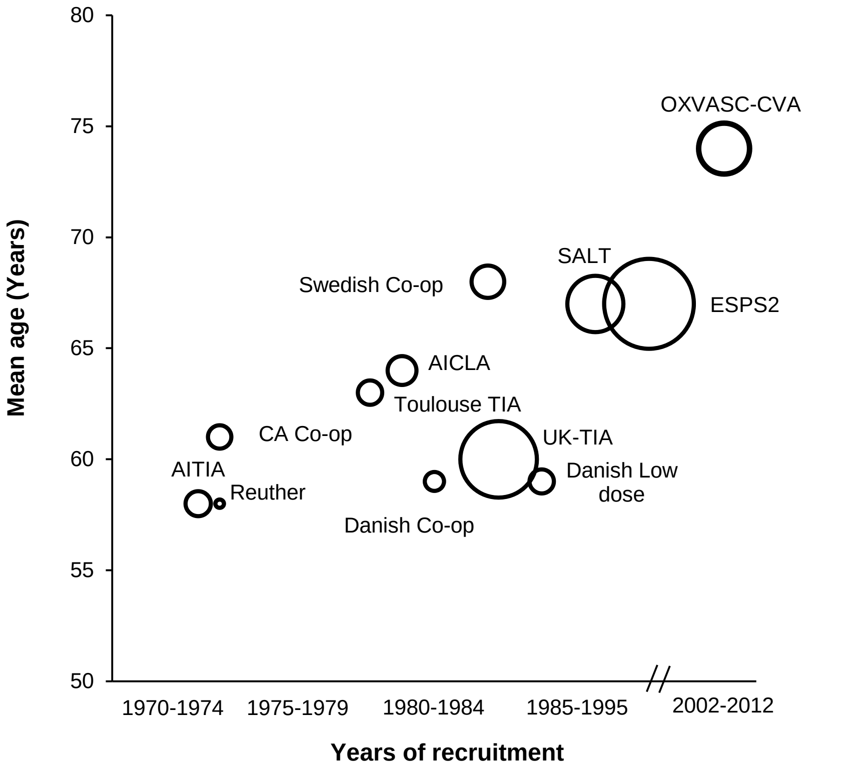

**Web appendix 2c. Age distribution of patients with TIA or ischaemic stroke in OXVASC and in aspirin trials of secondary prevention.**

UK TIA, ESPS1 and ESPS 2 are aspirin trials of secondary prevention. OXVASC included patients with a first-in-study-period myocardial infarction, transient ischaemic attack or ischaemic stroke from 2002 to 2012.

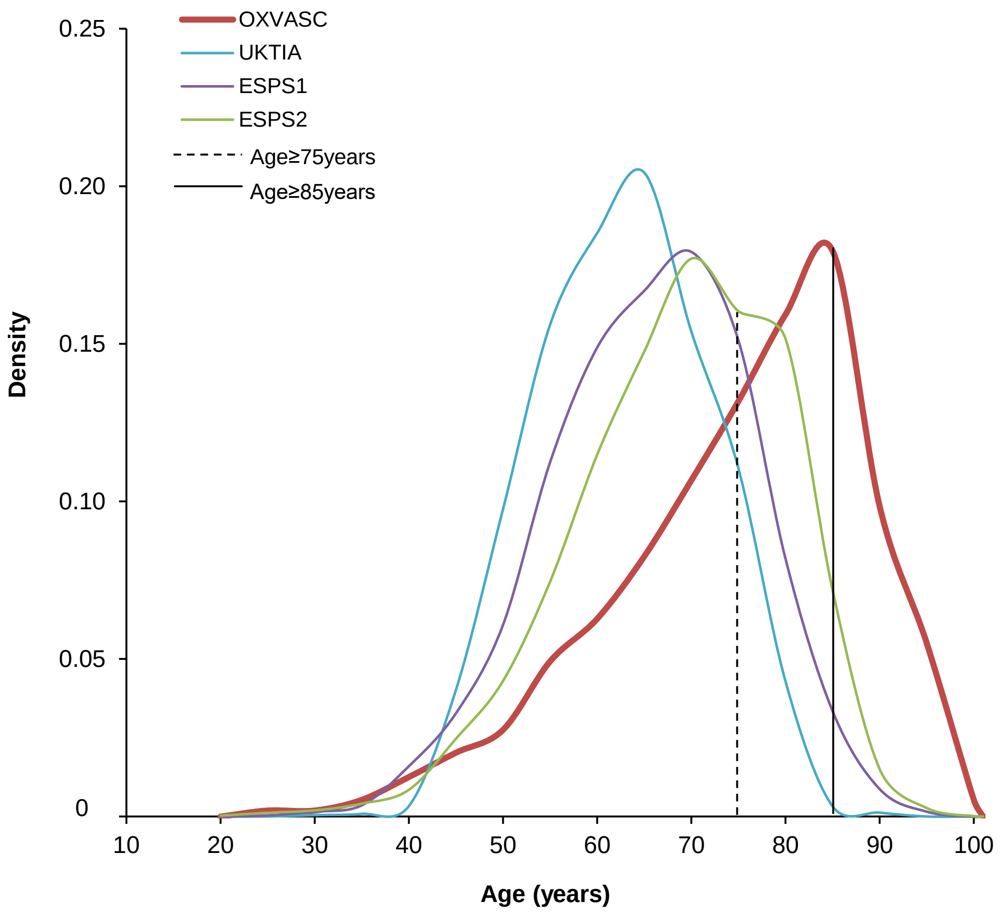

**Web appendix 3. Baseline characteristics of patients with myocardial infarction,TIA and ischaemic stroke in OXVASC who were initially treated with antiplatelet medication for secondary prevention stratified by age.** Categorial variables are given as n (%). $\chi^2$ test and t test were used to compare categorical and continuous variables between groups.

| | | Age group | | |
|---|---|---|---|---|
| | All (n=3166) | <75 (n=1584) | ≥75 (n=1582) | p |
| Mean (SD) Age (years) | 72.2 (13.4) | 61.4 (10.0) | 83.0 (5.4) | <0.0001 |
| Mean (SD) Weight (kg) | 74.7 (16.1) | 79.5 (17.2) | 69.8 (13.3) | <0.0001 |
| Female | 1449 (45.8) | 554 (35.0) | 895 (56.6) | <0.0001 |
| Acute event | | | | <0.0001 |
| Ischaemic stroke | 1177 (37.2) | 511 (32.3) | 666 (42.1) | |
| Transiet ischaemic attack | 895 (28.3) | 473 (29.9) | 422 (26.7) | |
| Non-ST elevation myocardial infarction | 703 (22.2) | 333 (21.0) | 370 (23.4) | |
| ST-elevation myocardial infarction | 391 (12.3) | 267 (16.9) | 124 (7.8) | |
| Prior events | | | | |
| Stroke | 277 (8.8) | 85 (5.4) | 192 (12.2) | <0.0001 |
| Transient ischaemic attack | 226 (7.1) | 74 (4.7) | 152 (9.6) | <0.0001 |
| Myocardial infarction | 369 (11.7) | 148 (9.3) | 221 (14.0) | <0.0001 |
| Peripheral vascular disease | 250 (7.9) | 104 (6.6) | 146 (9.2) | 0.0054 |
| Any previous vascular disease* | 902 (28.5) | 335 (21.1) | 567 (35.8) | <0.0001 |
| Hypertension | 1732 (54.8) | 754 (47.6) | 978 (61.9) | <0.0001 |
| Diabetes | 473 (15.0) | 251 (15.9) | 222 (14.1) | 0.16 |
| Hyperlipidaemia | 933 (29.5) | 486 (30.7) | 447 (28.3) | 0.14 |
| Current smoking† | 576 (18.3) | 456 (28.9) | 120 (7.7) | <0.0001 |
| Alcohol use >14units/week‡ | 452 (15.2) | 328 (21.8) | 124 (8.5) | <0.0001 |
| Anaemia | 663 (20.9) | 254 (16.0) | 409 (25.9) | <0.0001 |
| History of cancer | 383 (12.1) | 129 (8.2) | 254 (16.1) | <0.0001 |
| Chronic liver disease | 47 (1.5) | 30 (1.9) | 17 (1.1) | 0.06 |
| Renal failure | 45 (1.4) | 20 (1.3) | 25 (1.6) | 0.45 |
| History of peptic ulcer | 262 (8.3) | 98 (6.2) | 164 (10.4) | <0.0001 |
| Atrial fibrillation | 380 (12.0) | 79 (5.0) | 301 (19.1) | <0.0001 |
| Chronic heart failure | 308 (9.7) | 71 (4.5) | 237 (15.0) | <0.0001 |
| Premorbid antiplatelet use | 1279 (40.4) | 462 (29.2) | 817 (51.7) | <0.0001 |
| Premorbid PPI or H2-antagonist | 773 (24.4) | 323 (20.4) | 450 (28.4) | <0.0001 |
| Aspirin-based antiplatelet treatment post-event | 3027 (95.7%) | 1529 (96.6) | 1498 (94.8) | 0.0137 |
| Dual antiplatelet post-event†† | 1256 (39.7) | 733 (46.3) | 523 (33.1) | <0.0001 |

*History of vascular disease= history of stroke, TIA, myocardial infarction or peripheral vascular disease. †data missing in 6 patients aged <75years and 15 in patients aged ≥75years; ‡ data missing in 80 patients aged <75years and 122 in patients aged ≥75years. ††Dual antiplatelet treatment =aspirin+clopidogrel and only a small proportion of patients remained on dual antiplatelet treatment in the long-term. Aaemia=baseline haemoglobin <13g/l in men and <12g/l in women. Renal failure=glomerular filtration rate of <30ml/minute estimated using the Cockroft and Gault formula. TIA=Transient ischaemi attack. PPI=Proton Pump Inhibitors. H2-antagonist=Histamine 2 antagonist.

**Web appendix 4. Baseline charateristics of patients on premorbid gastric protection vs. those not on gastric protection.**
(Gastric protection=proton pump inhibitor or histamine 2 antagonist)

| | On gastric protection (n=773) | Not on gastric protection (n=2393) | p | Adjusted p† |
|---|---|---|---|---|
| Age (mean/SD) | 75.4/11.5 | 71.2/13.9 | <0.0001 | |
| Female | 381 (49.3) | 1068 (44.6) | 0.02 | 0.68 |
| | | | | |
| History of vascular disease* | 261 (33.8) | 641 (26.8) | 0.0002 | 0.03 |
| History of hypertension | 470 (60.8) | 1262 (52.8) | 0.0001 | 0.009 |
| History of diabetes | 138 (17.9) | 335 (14.0) | 0.009 | 0.008 |
| History of hyperlipidaemia | 267 (34.5) | 666 (27.9) | 0.0004 | 0.0002 |
| History of atrial fibrilation | 106 (13.7) | 275 (11.5) | 0.10 | 0.75 |
| History of cancer | 107 (13.8) | 276 (11.6) | 0.09 | 0.63 |
| History of chronic liver disease | 13 (1.7) | 34 (1.4) | 0.61 | 0.42 |
| History of renal failure | 14 (1.8) | 31 (1.3) | 0.30 | 0.35 |
| History of peptic ulcer | 124 (16.1) | 138 (5.8) | <0.0001 | <0.0001 |
| | | | | |
| Anaemia | 193 (25.0) | 470 (19.6) | 0.002 | 0.03 |
| Current smoking†† | 112 (14.5) | 464 (19.5) | 0.002 | 0.55 |
| Alcohol>14 units/week‡ | 94 (12.7) | 358 (16.1) | 0.03 | 0.48 |
| | | | | |
| Premorbid antiplatelet use | 378 (48.9) | 901 (37.7) | <0.0001 | 0.001 |
| Dual antiplatelet post-event | 305 (39.5) | 951 (39.8) | 0.87 | 0.28 |

Data are presented in number (%) unless otherwise stated. †Adjusted for age; *History of vascular disease= history of stroke, transient ischaemic attack, myocardial infarction or peripheral vascular disease. ††Data missing in 21 patients. ‡Data missing in 202 patients. Aaemia=baseline haemoglobin<13g/l in men and<12g/l in women. Renal failure=glomerular filtration rate of <30ml/minute estimated using the Cockroft and Gault formula.

**Web appendix 5. Sites of bleeding events requiring medical attention stratified by severity (CURE criteria).**

| Bleeding site | Severity of bleeding | | | | Total |
|---|---|---|---|---|---|
| | Significant non-major | Major non-life threatening | Life threatening | Fatal | |
| Intracranial (n,%) | 0 (0) | 0 (0) | 21 (26.3) | 24 (54.5) | 45 (11.1) |
| Intracerebral (n,%) | 0 (0) | 0 (0) | 11 (13.8) | 17 (38.6) | 28 (6.9) |
| Subdural (n,%) | 0 (0) | 0 (0) | 6 (7.5) | 4 (9.1) | 10 (2.5) |
| Subarachnoid (n,%) | 0 (0) | 0 (0) | 4 (5.0) | 3 (6.8) | 7 (1.7) |
| Gastrointestinal (n,%) | 105 (48.2) | 47 (74.6) | 47 (58.8) | 19 (43.2) | 218 (53.8) |
| Upper-gastrointestinal (n,%) | 65 (29.8) | 38 (60.3) | 42 (52.5) | 17 (38.6) | 162 (40.0) |
| Lower-gastrointestinal (n,%) | 40 (18.3) | 9 (14.3) | 5 (6.3) | 2 (4.5) | 56 (13.8) |
| Epistaxis (n,%) | 42 (19.3) | 1 (1.6) | 1 (1.3) | 0 (0) | 44 (10.9) |
| Genitourinary (n,%) | 44 (20.2) | 7 (11.1) | 2 (2.5) | 0 (0) | 53 (13.1) |
| Other (n,%) | 27 (12.4) | 8 (12.7) | 9 (11.3) | 1 (2.3) | 45 (11.1) |
| Total (n, %total bleed) | 218 (53.8) | 63 (15.5) | 80 (19.8) | 44 (10.9) | 405 |

**Web appendix 6. Long-term risks of bleeding events requiring medical attention stratified by severity, site of bleeding and index event type.**

(MI: myocardial infarction, CVA: cerebrovascular disease)

**A. Any bleed**

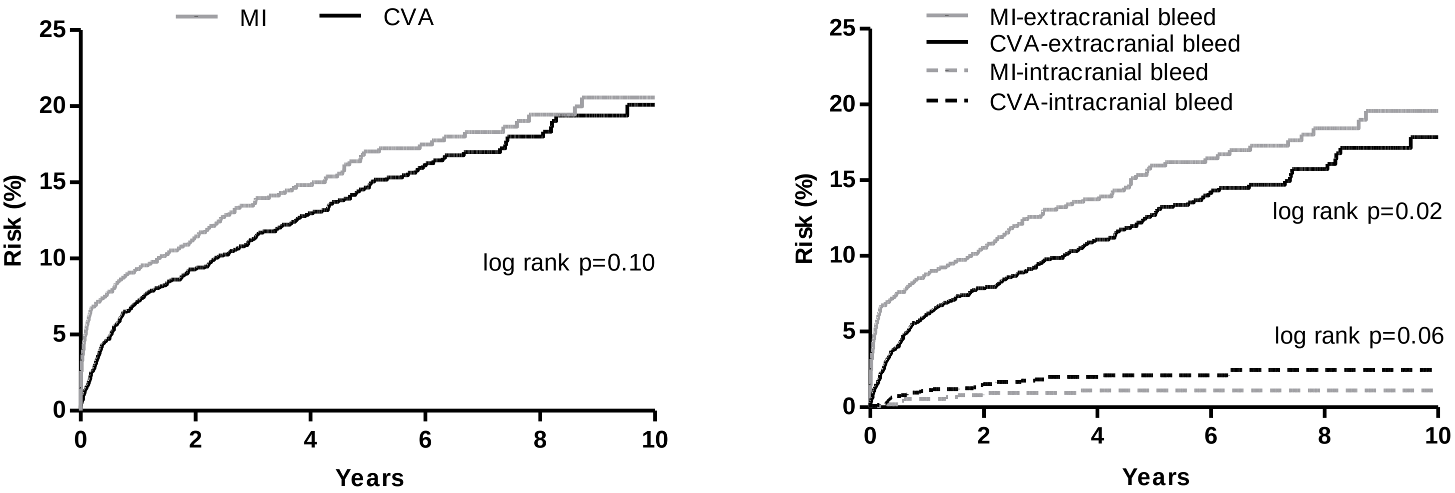


**B. Any major bleed**

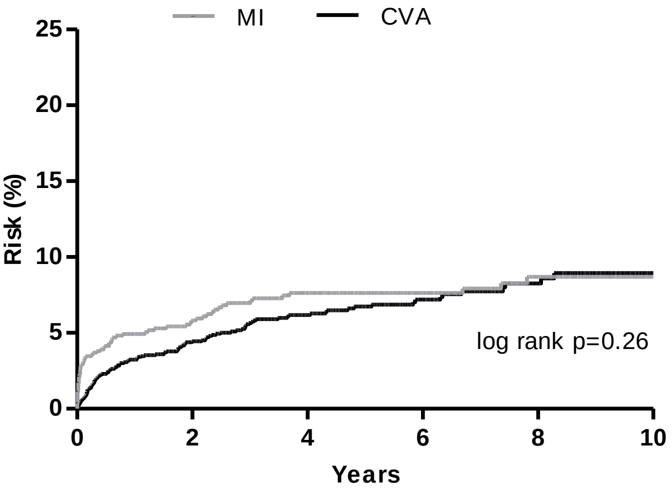


**C. Any significant non-major bleed**

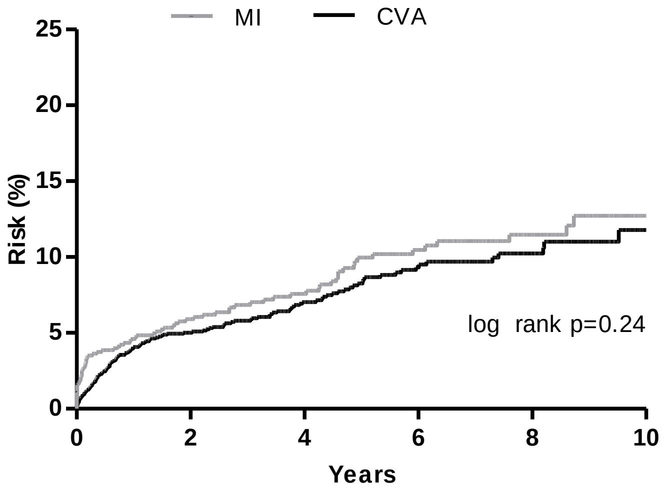

**Web appendix 7. Age-specific risks of bleeding events requiring medical attention stratified by sites and severity of bleeding.** (GI=gastrointestinal)

### All bleeding events

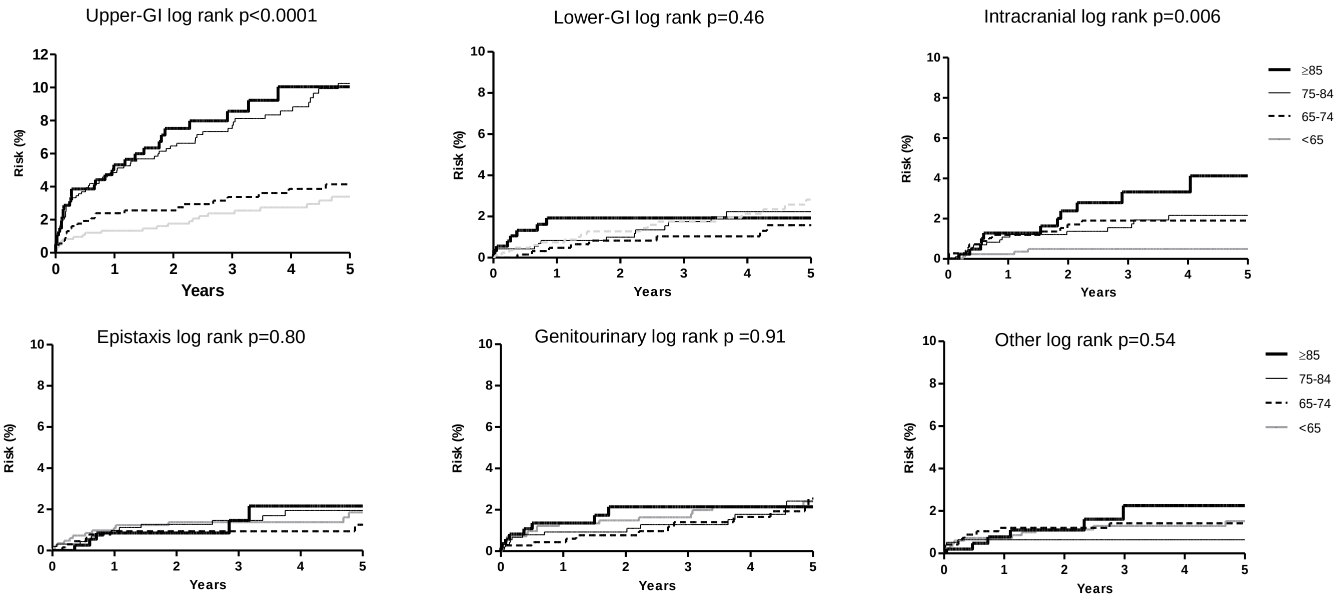


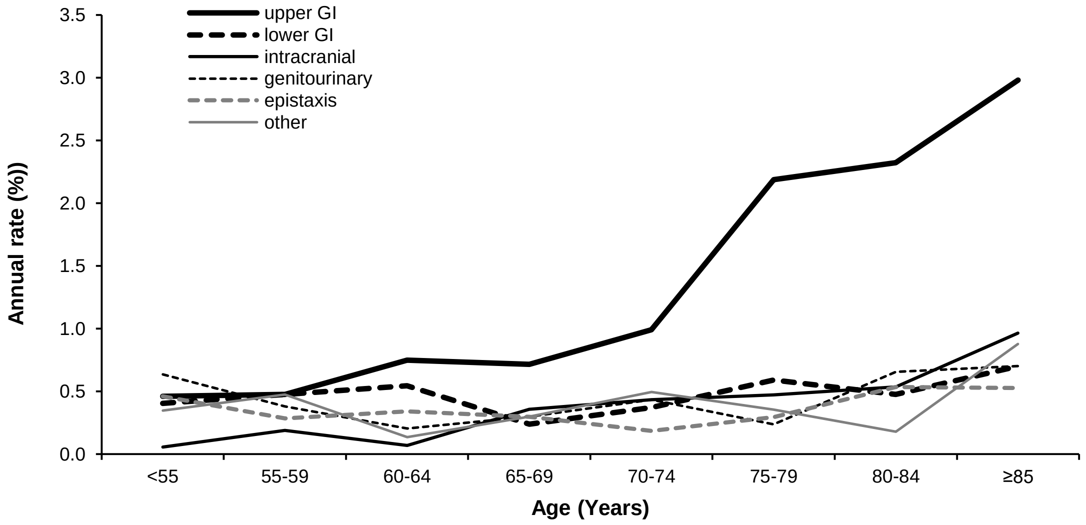


### Major bleed

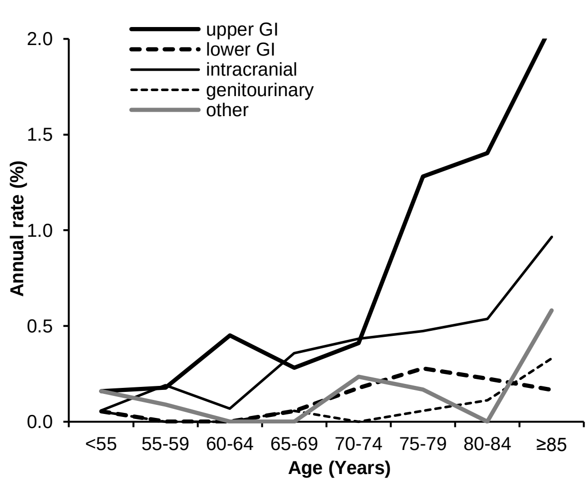


### non-major bleed

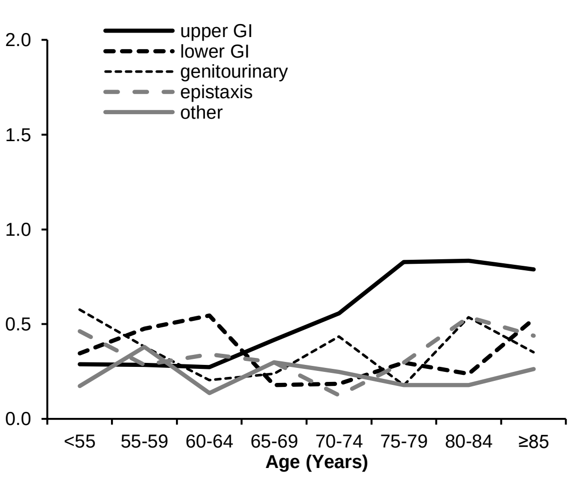

## Web appendix 8. Risks of major bleeding vs. recurrent ischaemic vascular events in aspirin-controlled trials for secondary prevention of stroke.

| Names | Patient group | Aspirin (mg/day) | Aspirin group (N) | Mean age | Mean follow-up | Patient-years | Major bleed† | Recurrent ischaemic vascular events | | Bleed / ischaemic |
|---|---|---|---|---|---|---|---|---|---|---|
| | | | | | | | N (risk %yr) | Definication of recurrent ischaemic vascular events | N (risk %yr) | Ratio (excess risk of ischaemic/bleed %) |
| **Aspirin vs. placebo** | | | | | | | | | | |
| AITIA (medical arm)[e12] | TIA | 1300 | 88 | 58 | 2 years | 176 | 2 (1.1) | Ischaemic stroke and MI | 12 (6.8) | 0.2:1 (400%) |
| UK-TIA (low dose)[e13] | TIA and minor stroke | 300 | 806 | 60 | 4 years | 3224 | 17 (0.5) | Stroke/MI/vascular death | 178 (5.5) | 0.1:1 (900%) |
| UK-TIA (high dose)[e13] | TIA and minor stroke | 1200 | 815 | 60 | 4 years | 3260 | 26 (0.8) | Stroke/MI/vascular death | 176 (5.4) | 0.1:1 (900%) |
| Dutch TIA (low dose)[e14] | TIA and minor stroke | 30 | 1555 | 63† | 2.6 years | 4665 | 40 (0.9) | Stroke/MI/vascular death | 228 (4.9) | 0.2:1 (400%) |
| Dutch TIA (high dose)[e14] | TIA and minor stroke | 283 | 1576 | 63‡ | 2.6 years | 4728 | 53 (1.1) | Stroke/MI/vascular death | 240 (5.1) | 0.2:1 (400%) |
| SALT[e15] | TIA, minor stroke and retinal artery occlusion | 75 | 676 | 67 | 32 months | 1803 | 20 (1.1) | Ischaemic stroke and MI/SCD | 136 (7.5) | 0.2:1 (400%) |
| Swedish co-op[e16] | Ischaemic stroke | 1500 | 253 | 68 | 2 years | 506 | 6 (1.2) | Ischaemic stroke and MI | 50 (9.9) | 0.1:1 (900%) |
| ESPS-2[e17] | TIA and ischaemic stroke | 50 | 1649 | 67 | 2 years | 3298 | 53 (1.6) | Ischaemic stroke/MI/sudden death | 266 (8.1) | 0.2:1 (400%) |
| **Aspirin vs. other antiplatelet agents currently in secondary prevention of stroke** | | | | | | | | | | |
| ESPIRIT[e18] | TIA and minor stroke | 30-325 | 1376 | 63 | 3.5 years | 4495 | 53 (1.2) | Non-fatal ischaemic stroke, non-fatal MI or non-haemorrhagic vascular death | 174 (3.9) | 0.3:1 (233%) |
| SPS-3[e19] | Recent lacunar infarct | 325 | 1503 | 63 | 3.4 years | 5110 | 56 (1.1) | Ischaemic stroke and MI | 162 (3.2) | 0.3:1 (233%) |
| CAPRIE[e20] | Ischaemic stroke, MI or PVD | 325 | 9546 | 63 | 1.9 years | 18233 | 149 (0.8) | Ischaemic srtoke and MI | 922 (5.1) | 0.2:1 (400%) |
| **Aspirin vs. Warfain** | | | | | | | | | | |
| WARSS[e21] | Ischaemic stroke | 325 | 1103 | 63 | 2 years | 2206 | 30 (1.4) | Ischaemic stroke | 123 (5.6) | 0.2:1 (400%) |
| WASID[e22] | TIA and stroke patients with 50-99%intracranial stenosis | 1300 | 280 | 63 | 1.8 years | 504 | 9 (1.8) | Ischaemic stroke and MI | 64 (12.7) | 0.1:1 (900%) |
| SPIRIT[e23] | TIA and minor stroke | 30 | 665 | 63 | 14 months | 776 | 6 (0.8) | Non-fatal ischaemic stroke or non-fatal MI or non-haemorrhagic vascular death | 27 (3.5) | 0.2:1 (400%) |

† not reported, 54.5% >65 years; ‡ not reported, 52.6% >65yrs; TIA=Transient ischaemic attack, MI=myocardial infarction, SCD=sudden cardiac death, PVD=Peripheral vascular disease.

**†Defination of major bleed in the trials**

| Trial names | Definition of major bleeding |
|---|---|
| AITIA (medical arm) | Collected from direct description of adverse events |
| UK TIA (low dose) | Intracranial haemorrhage; GI bleed that was fatal or was admitted to hospital |
| UK TIA (high dose) | Intracranial haemorrhage; GI bleed that was fatal or was admitted to hospital |
| Dutch TIA (low dose) | Fatal or if a hospital visit and treatment were necessary. Epistaxis, easy bruising and melena were regarded as minor if no treatment was required |
| Dutch TIA (high dose) | Fatal or if a hospital visit and treatment were necessary. Epistaxis, easy bruising and melena were regarded as minor if no treatment was required |
| SALT | Severe bleeding or causing discontinuation of study drug (gastrointestinal/intracranial/other) |
| Swedish co-op | Intracerebral haemorrhage and other severe haemorrhage |
| ESPS-2 | Moderate (requiring specific treatment but no transfusion)/severe bleeding (requiring blood transfusion) of any site |
| ESPIRIT | All intracranial bleeding, any fatal bleeding or any bleeding requiring hospital admission |
| SPS-3 | All intracranial bleeding, and extracranial haemorrhage that is serious or life-threatening requiring transfusion or surgery or resulting in permeant functional sequelae or death |
| CAPRIE | Any severe bleeding |
| WARSS | Intracranial, intraspinal, intracerebral, subarachnoid, subdural or epidural haemorrhage or any other bleeding event requiring transfusion |
| WASID | Intracranial haemorrhage or systemic haemorrhage requiring hospitalization, blood transfusion or surgery |
| SPIRIT | Intracranial bleeding, fatal bleeding and any bleeding requiring hospitalization irrespective of interventions |

**Web appendix 9. Annual rates of major bleeding in aspirin-controlled trials for secondary prevention of stroke vs. OXVASC (cerebrovascular cohort).**

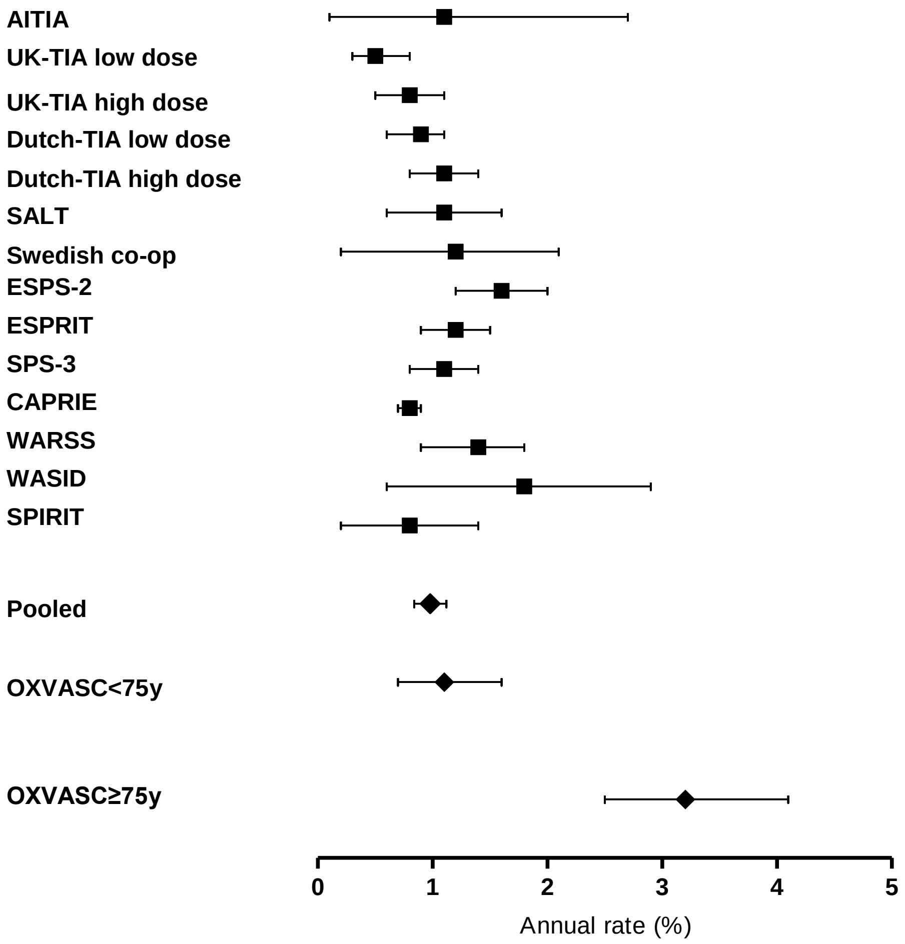

**Web appendix 10. Annual rates of life-threatening and fatal bleeding stratified by age (left panels) and the distributions of bleeding of different severity by age (right panels).** (A: aspirin, C: clopidogrel)

Annual rate derived as N/100 patient-years

**Major bleed**

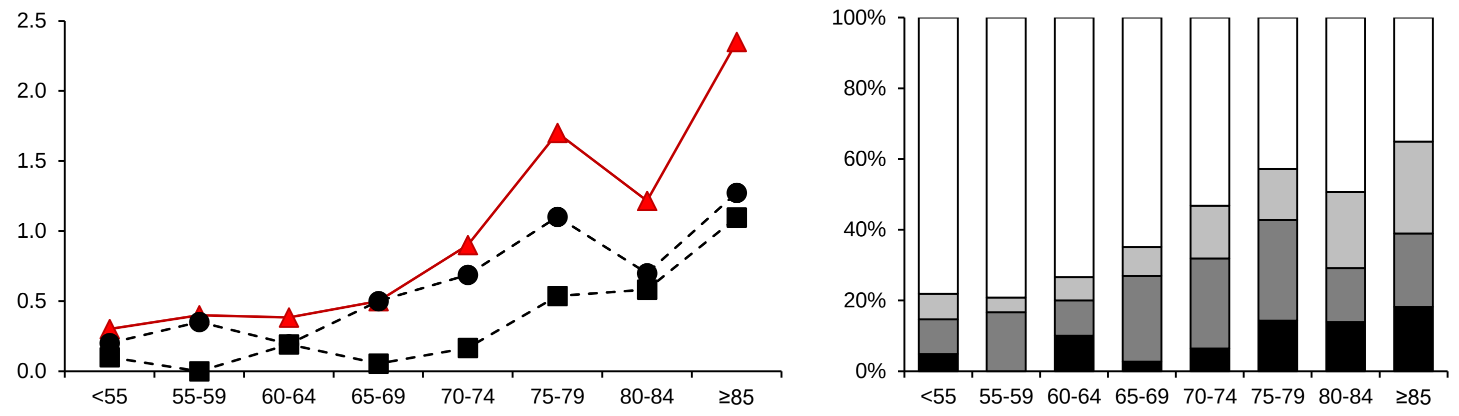


**Major bleed still on antiplatelet agent**

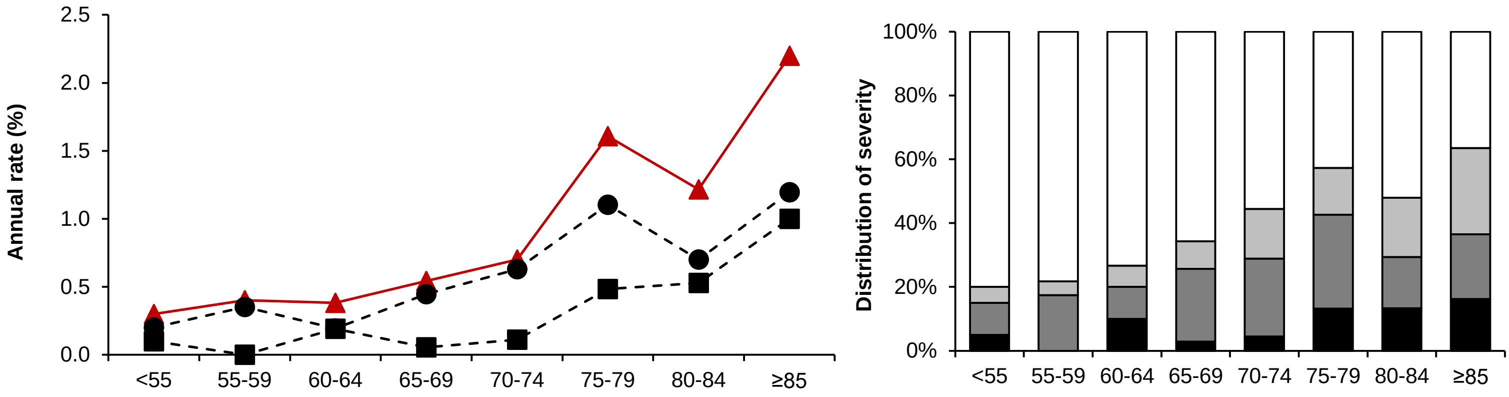


**Major bleed on antiplatelet agent (excluding A+C)**

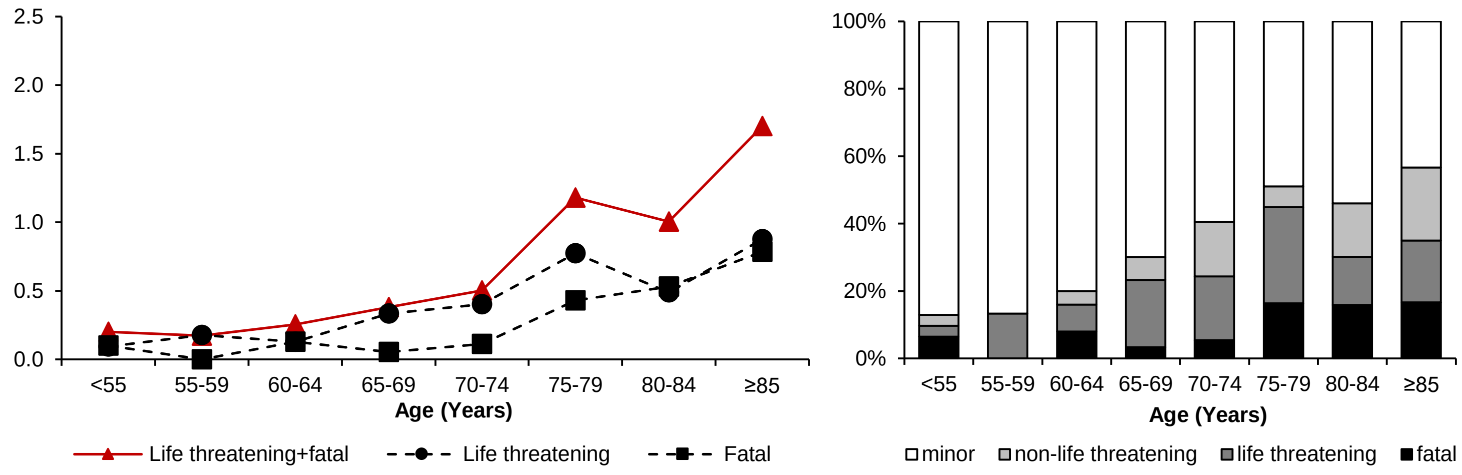

**Web appendix 11. Types of the antiplatelet agents patients were taking immediately prior to the bleeding events that required medical attention stratified by age.**

| | <75 years (n=179) | ≥75 years (n=226) | Total |
|---|---|---|---|
| No antiplatelet | 6 (3.4) | 9 (4.0) | 15 (3.7) |
| Aspirin | 114 (63.7) | 140 (61.9) | 254 (62.7) |
| Clopidogrel | 9 (5.0) | 15 (6.6) | 24 (5.9) |
| Dipyridamole | 1 (0.6) | 0 (0) | 1 (0.2) |
| A+C | 28 (15.6) | 22 (9.7) | 50 (12.3) |
| A+D | 12 (6.7) | 13 (5.8) | 25 (6.2) |
| A+heparin | 2 (1.1) | 11 (4.9) | 13 (3.2) |
| A+C+heparin | 5 (2.8) | 15 (6.6) | 20 (4.9) |
| A+rt-PA | 2 (1.1) | 1 (0.4) | 3 (0.7) |

*A=Aspirin, C=Clopidogrel, D=Dipyridamole, rt-PA= Recombinant tissue plasminogen activator.

**Web appendix 12. Risks of major bleeding in patients aged ≥75 years vs. patients aged <75 years stratified by site of bleeding.**

| | <75 years | | ≥75 years | | | |
|---|---|---|---|---|---|---|
| | Risk (per 1000 patient-years) | Cumulated 10-year risk (%) | Risk (per 1000 patient-years) | Cumulated 10-year risk (%) | **HR (95%CI)** | **p** |
| **Major GI bleed** | 3.60 | 2.37 | 17.56 | 11.46 | 4.00 (2.62-6.12) | <0.0001 |
| Major upper-GI | 2.98 | 1.93 | 15.26 | 9.16 | 4.13 (2.60-6.57) | <0.0001 |
| Major lower-GI | 0.62 | 0.45 | 2.30 | 2.53 | 3.34 (1.15-9.76) | 0.03 |
| **Intracranial bleed** | 2.08 | 1.28 | 5.64 | 3.13 | 2.21 (1.21-4.05) | 0.01 |
| Intracerebral | 0.99 | 0.65 | 4.18 | 2.29 | 3.38 (1.48-7.70) | 0.004 |
| Subarachnoid | 0.50 | 0.29 | 0.63 | 0.37 | 1.03 (0.23-4.63) | 0.97 |
| Subdural | 0.62 | 0.36 | 1.05 | 0.53 | 1.33 (0.39-4.62) | 0.65 |
| **Major other** | 1.37 | 0.77 | 3.76 | 2.21 | 2.10 (0.99-4.45) | 0.06 |

*GI=gastrointestinal

**Web appendix 13. Risks of major bleeding in patients aged ≥75 years vs. patients aged <75 years stratified by type of the index event.**

| | Ischaemic stroke/TIA cohort | | Myocardial infarction cohort | |
|---|---|---|---|---|
| | HR (95%CI) | p | HR (95%CI) | p |
| Major bleeds | 3.09 (2.06-4.62) | <0.0001 | 3.27 (1.99-5.37) | <0.0001 |
| Upper-GI | 5.43 (2.73-10.81) | <0.0001 | 3.48 (1.83-6.61) | 0.0001 |
| Intracranial bleed | 2.06 (1.04-4.07) | 0.04 | 2.20 (0.59-8.23) | 0.24 |
| Other | 2.06 (0.94-4.52) | 0.07 | 3.44 (1.27-9.32) | 0.02 |

*GI=gastrointestinal

**Web appendix 14. Reasons for new or worsening of disability attributable to the bleeding events.**

Number (bleed severity: life threatening/non-life threatening major bleed)

| | **Intracranial bleed** (n=11) | **Extracranial bleed** (n=50) |
|---|---|---|
| **Followed by ischaemic stroke** | 0 | 4 (1/3) |
| **Blindness** | 0 | 2 (1/1) |
| **Cognitive decline** | 0 | 2 (0/2) |
| **Reduced mobility** | 3 (3/0) | 37 (17/20) |
| General decline | 1 (1/0) | 3 (0/3) |
| Symptomatic anaemia (e.g. short of breath) | 0 | 6 (4/2) |
| New or worsening congestive cardiac failure/pulmonary oedema | 0 | 15 (7/8) |
| Severe infection or acute renal failure | 0 | 5 (3/2) |
| Worsening of other co-morbidities | 2 (2/0) | 8 (3/5) |
| **Subsequent cancer diagnosis and under palliative care** | 0 | 4 (3/1) |
| **Intracranial bleed associated neurological deficit** | 8 (8/0) | 1 (0/1) |

*CCF=congestive cardiac failure, ARF=acute renal failure

**Web appendix 15. Reasons for death attributable to the bleeding events.**

| | Intracranial bleed (n=24) | Extracranial bleed (n=20) |
|---|---|---|
| **Followed by ischaemic stroke** | 0 | 1 |
| **Reduced mobility** | 5 | 5 |
| New or worsening chronic cardiac failure/pulmonary oedema | 0 | 1 |
| Severe infection or acute renal failure | 2 | 3 |
| Worsening of other co-morbidities | 3 | 1 |
| **Subsequent cancer diagnosis and under palliative care** | 0 | 2 |
| **Herniation-massive intracranial bleed with midline shift and oedema** | 19 | 0 |
| **Bleeding associated hypovolemic/cardiac shock** | 0 | 12 |

**Web appendix 16. Long-term risks of bleeding requiring medical attention stratified by the number of the initial antiplatelet treatment.**

(Mono: monotherapy, including also aspirin plus dipyridamole; Dual: dual therapy with aspirin and clopidogrel)

**A. Any bleed**

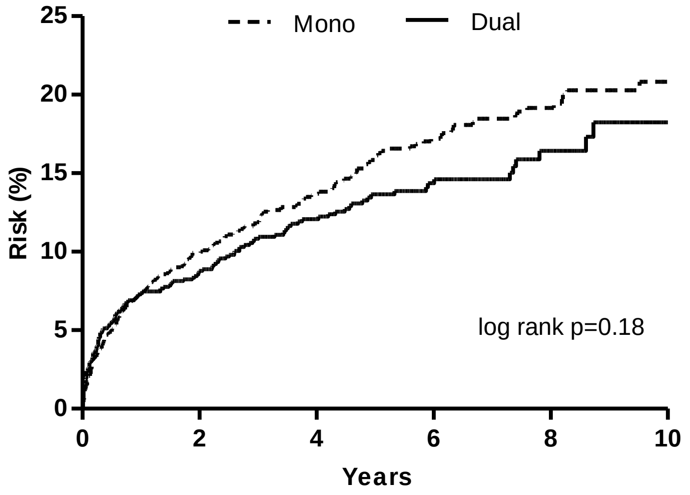


**B. Major bleed**

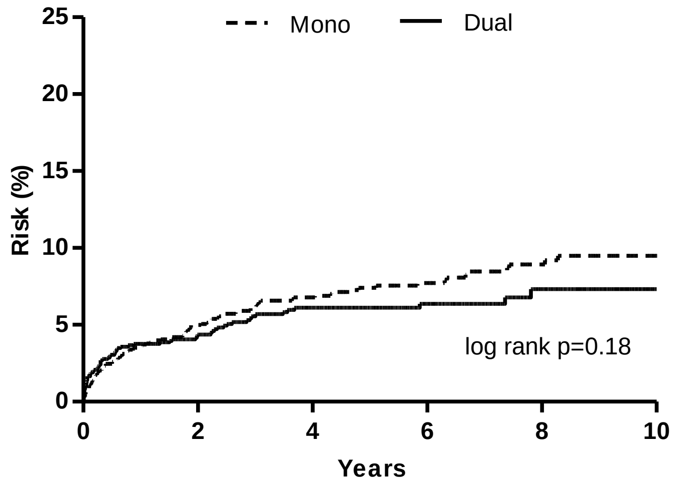

**Web appendix 17. Sensitivy analyses of the long-term risks of bleeding events requiring medical attention stratified by severity, site of bleeding and the index event type excluding the first 90 days.**
(MI: myocardial infarction, CVA: cerebrovascular disease)

**A. Any bleed**

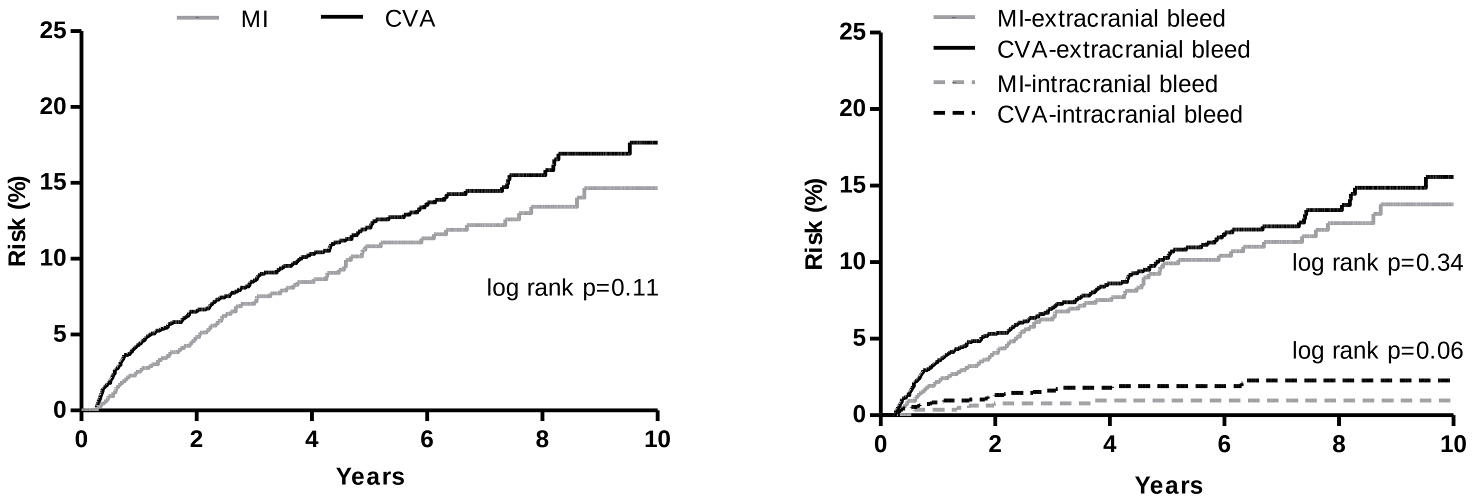


**B. Any major bleed**

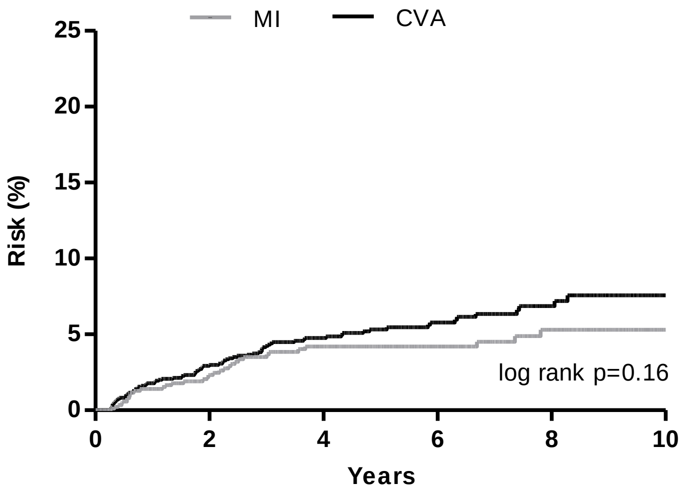


**C. Any significant non-major bleed**

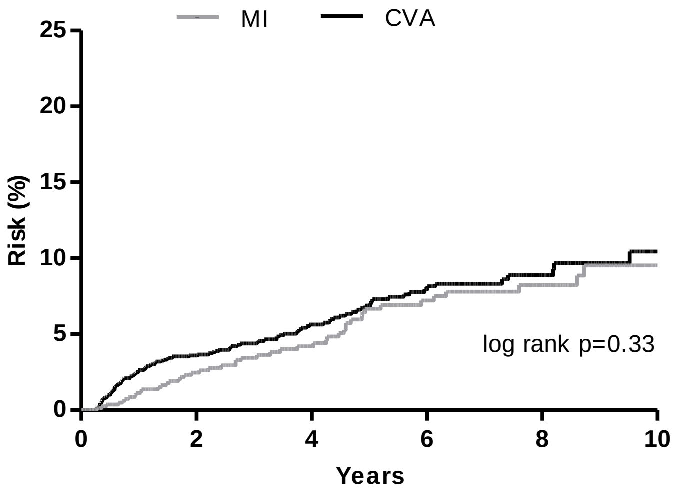

**Web appendix 18. Time-course of risks of major bleeding events in patients ≥75 years versus patients <75 years.**

HR=hazard ratio, CI=confidence interval;

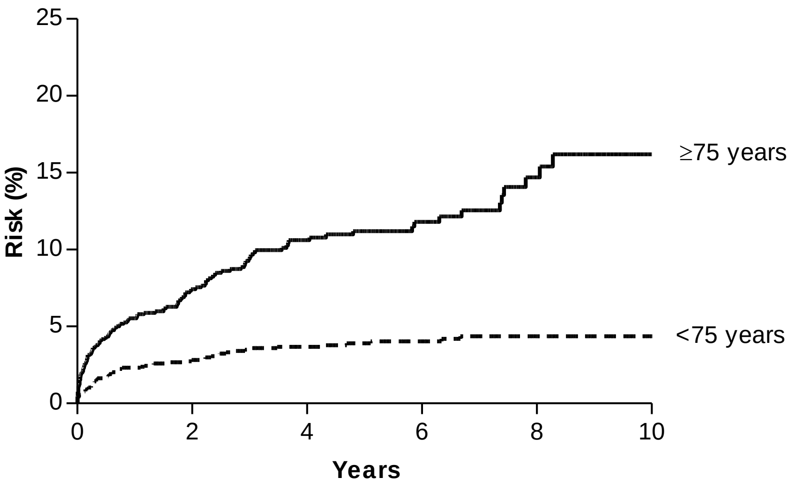


| Number at risk | | | | | | |
|---|---|---|---|---|---|---|
| <75 years | 1584 | 1243 | 930 | 638 | 371 | 118 |
| ≥75 years | 1582 | 844 | 504 | 274 | 125 | 31 |

| | **Year 1** | **Year 1-4** | **Year 4-10** |
|---|---|---|---|
| | Event/patient years | Event/patient years | Event/patient years |
| <75 years | 35/1,465 | 17/3,488 | 5/3,097 |
| ≥75 years | 74/1,186 | 43/2,285 | 13/1,313 |
| HR (95%CI) | 2.45 (1.64-3.67) | 3.80 (2.17-6.68) | 6.11 (2.17-17.18) |

**Web appendix 19. Age-specific time-course of major bleeding stratified by premorbid antiplatelet drug use at baseline (A) and in patients who had been stable on antiplatelet treatment for one year (B).**

Panel A shows the age-specific time-couse of major bleeding in patients on premorbid antiplatelet drug (A1) and in those not on premorbid antiplatelet drug (A2). Panel B shows the age-specific time-course of major bleeding in patients who had been on antiplatelet treatment for one year after acute TIA, ischaemic stroke or myocardial infarction (i.e. excluding patients who had major bleeding events or had died before one year). Age is classified as the age of the patient at one year.

## A. All patients at baseline

### A1. On premorbid antiplatelet drug

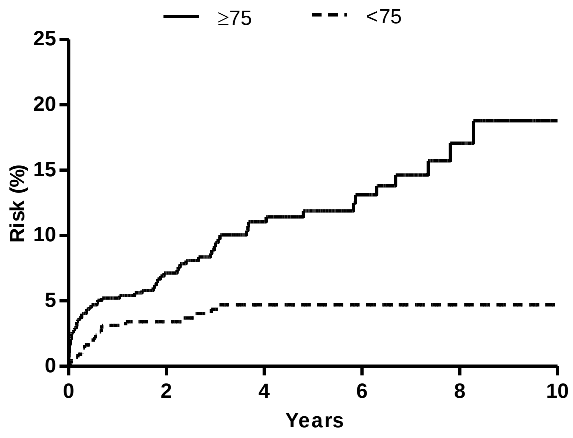


### A2. Not on premorbid antiplatelet drug

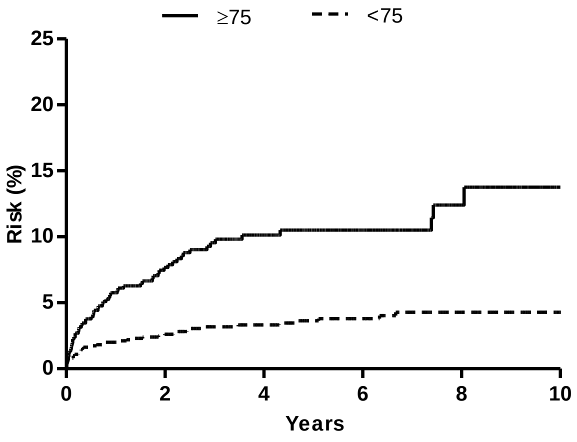


## B. Excluding patients who had had a major bleed on antiplatelet treatment during the first year of follow-up

### B1. Major bleed

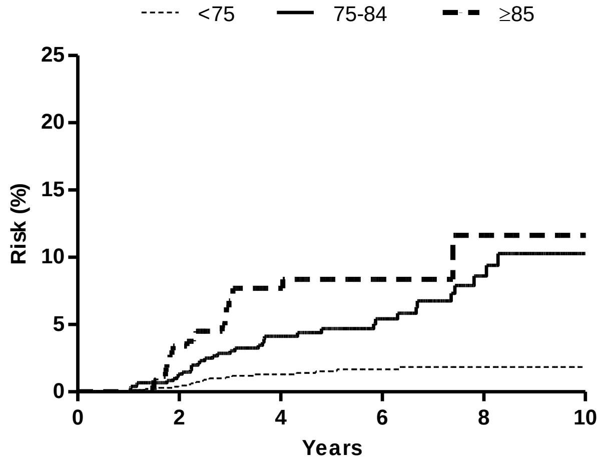


### B2. Major upper GI bleed

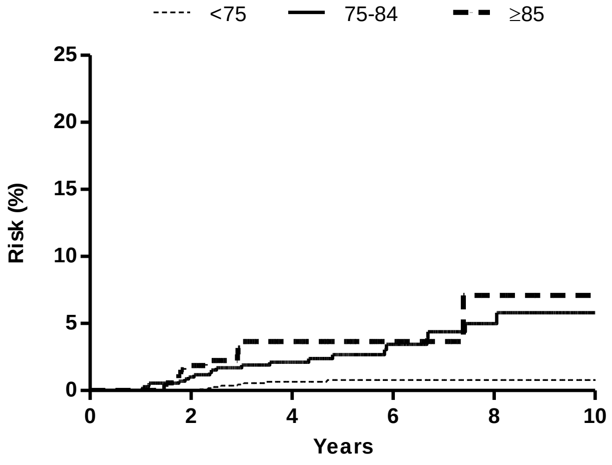

**Web appendix 20. Time-course of major bleeding in patients aged ≥ 75 years vs. patients aged <75 years stratified by site of bleeding.** (GI=gastrointestinal)

**A. Upper-GI bleed**

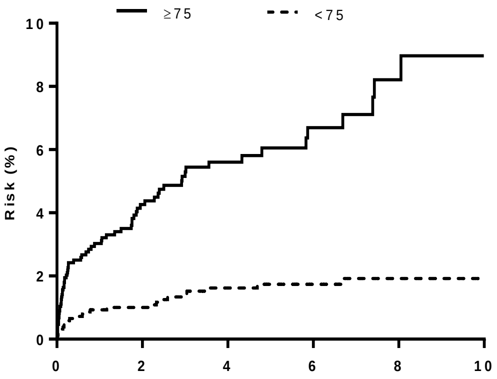


**B. Lower-GI bleed**

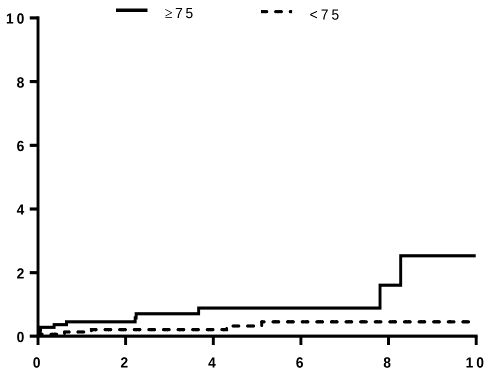


**C. Intracranial bleed**

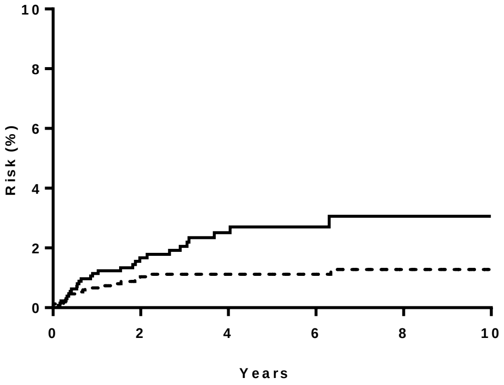


**D. Other bleed**

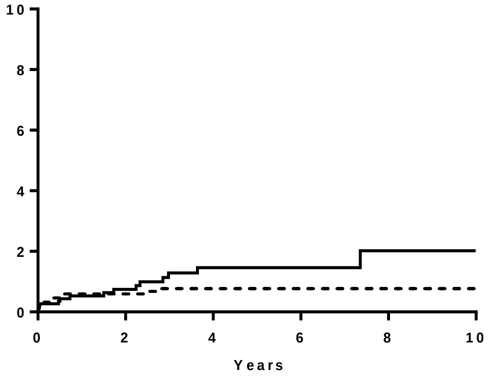

**Web appendix 21. Cumulative 5-year risk of major bleeds and major upper-GI bleeds in OXVASC stratified by the REACH bleeding risk score and by age.**

| REACH score | N | Major bleeds | Major upper-GI bleeds |
|---|---|---|---|
| | | Event (%) | Event (%) |
| **<75 years** | | | |
| 0-6 | 823 | 18 (2.6) | 6 (0.9) |
| 7-8 | 482 | 19 (4.5) | 9 (2.3) |
| ≥9 | 279 | 17 (7.1) | 8 (3.4) |
| **≥75 years** | | | |
| 0-6 | 0 | - | - |
| 7-8 | 269 | 18 (9.0) | 11 (5.7) |
| 9-10 | 678 | 49 (10.1) | 27 (5.7) |
| >10 | 638 | 53 (14.0) | 29 (7.1) |

**Web appendix 22. Observed 5-year risks of major bleeding versus recurrent ischaemic events stratified by age and by the REACH bleeding risk score.**
(Patients with history of atrial fibrillation on antiplatelet treatment excluded; data presented with 95%CI)

**Excluding recurrent ischaemic or bleeding events within 90 days post index events** | **All events included**

**Age**

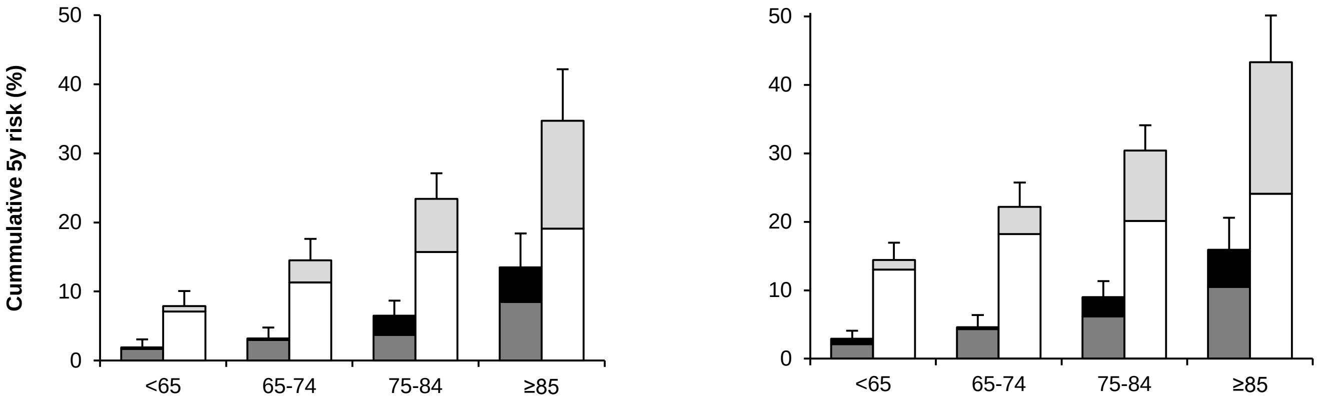


**REACH score**

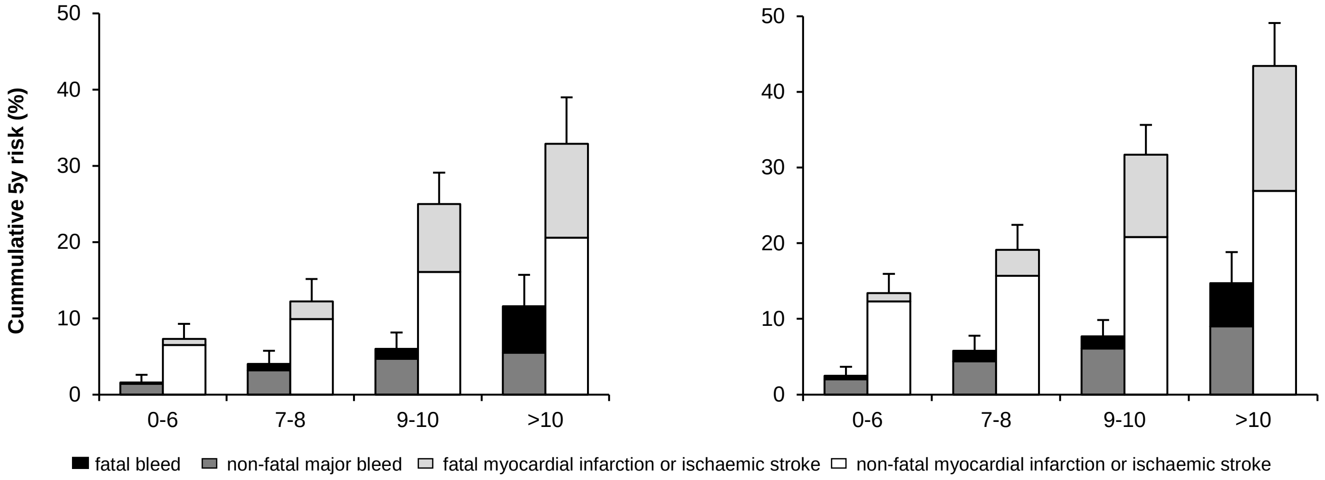

**Web appendix 23. Observed age-specific 5-year risks of major bleeding vs. ischaemic events in OXVASC stratified by the type of the index event.**

(Patients with history of atrial fibrillation on antiplatelet treatment excluded;MI=myocardial infarction;data presented with 95%CI)

**Excluding recurrent ischaemic or bleeding events within 90 days post index events**

**All events included**

**Whole cohort**

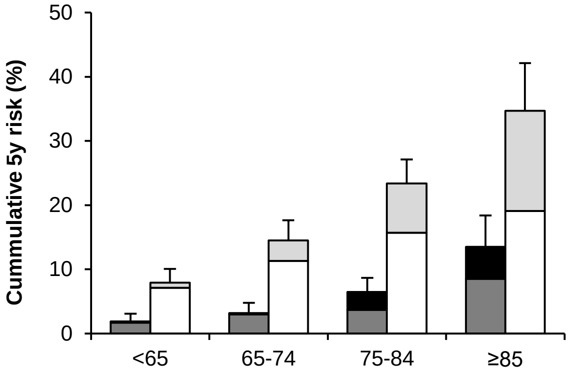


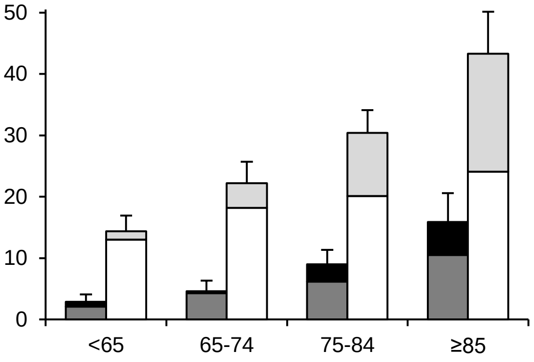


**Cerebrovascular cohort**

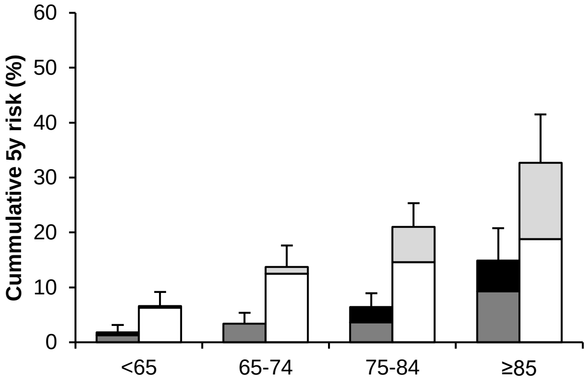


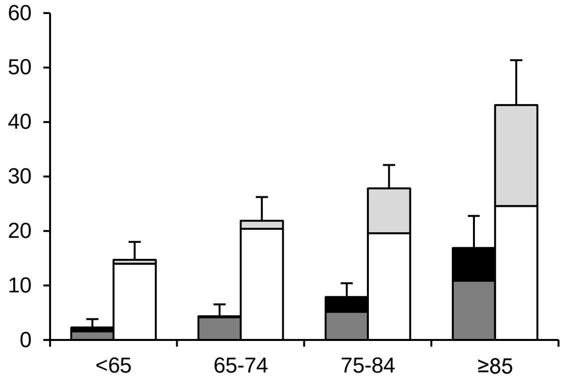


**MI cohort**

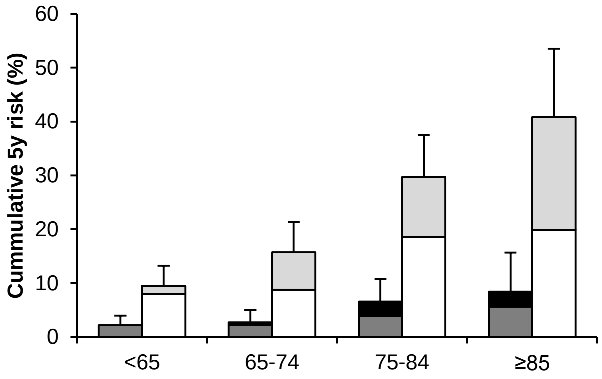


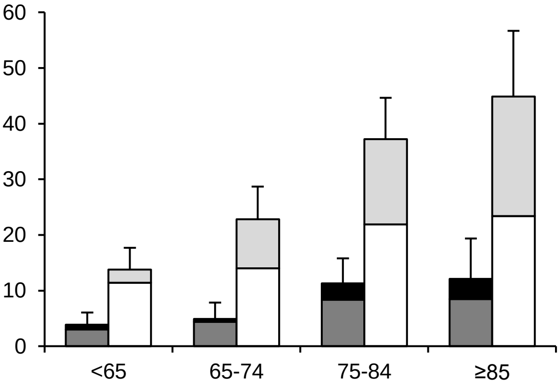


■fatal bleed ■ non-fatal major bleed □ fatal myocardial infarction or ischaemic stroke □ non-fatal myocardial infarction or ischaemic stroke

**Web appendix 24. Estimated 5-year risk of major bleeding attributable to antiplatelet treatment vs. ischaemic events prevented by antiplatelet treatment stratified by age.**

(Patients with history of atrial fibrillation on antiplatelet treatment excluded;MI=myocardial infarction;data presented with 95%CI)

**Excluding recurrent ischaemic or bleeding events within 90 days post index events** | **All events included**

**Whole cohort**

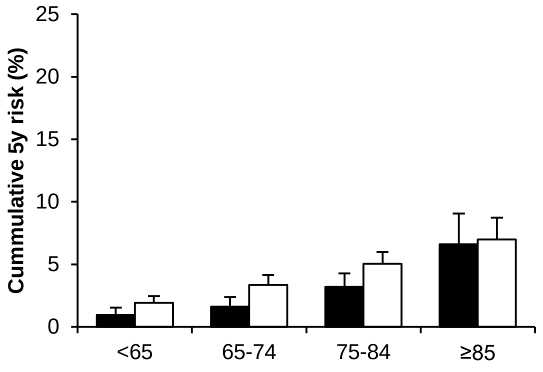


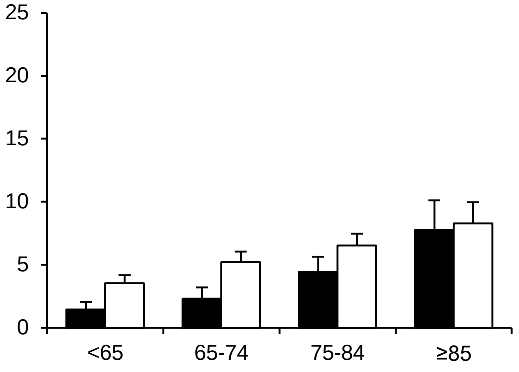


**Cerebrovascular cohort**

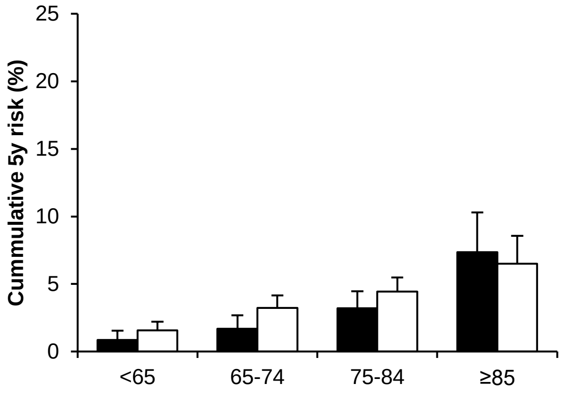


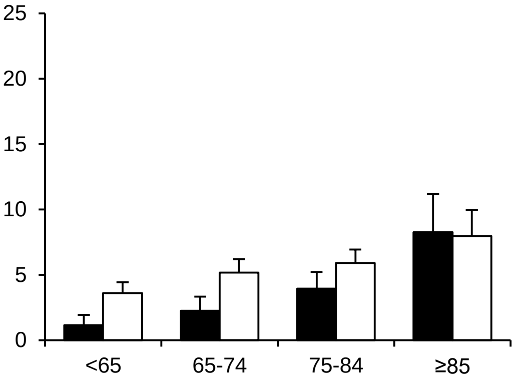


**MI cohort**

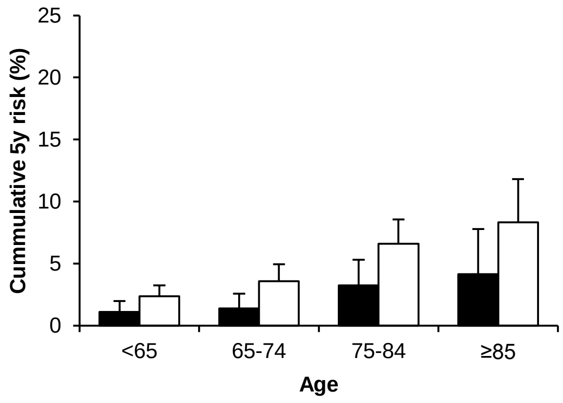


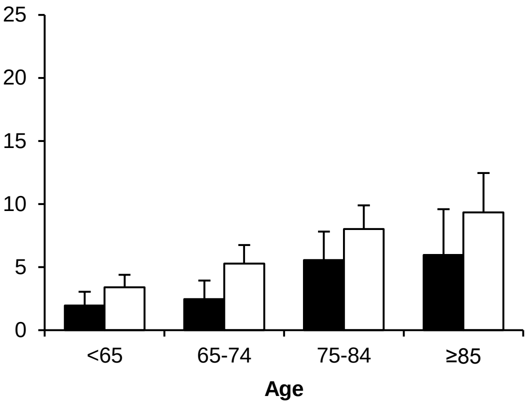


■ major bleed　　□ myocardial infarction, sudden cardiac death or non-fatal ischaemic stroke

**Web appendix 25. Sensitivity analyses censoring at a composite outcome of major bleeding events or recurrent ischaemic events for the observed age-specific 5-year risks of major bleeding vs. ischaemic events.** (Patients with history of atrial fibrillation on antiplatelet treatment excluded;MI=myocardial infarction;data presented with 95%CI)

**Excluding recurrent ischaemic or bleeding events within 90 days post index events**

**All events included**

**Whole cohort**

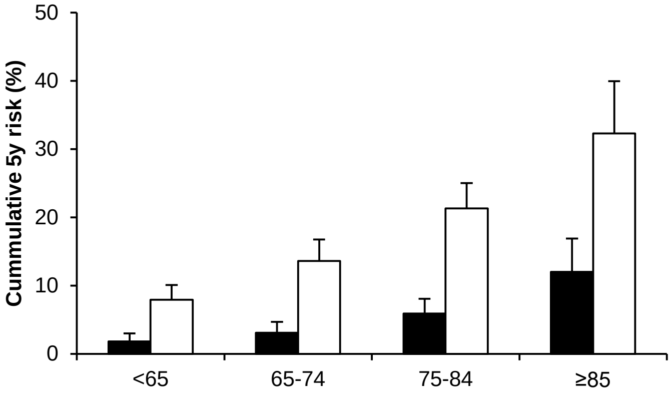


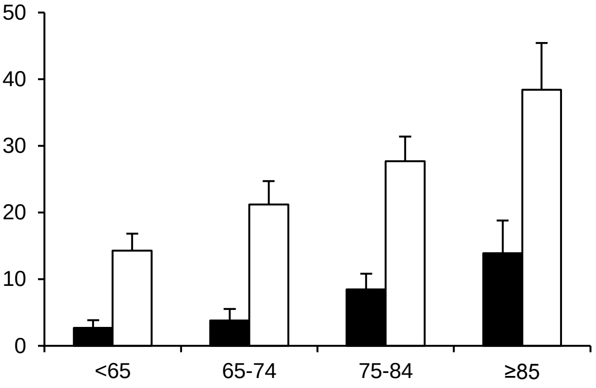


**Cerebrovascular cohort**

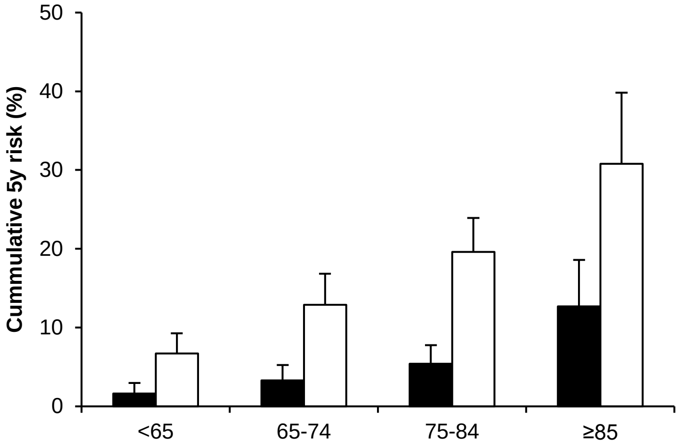


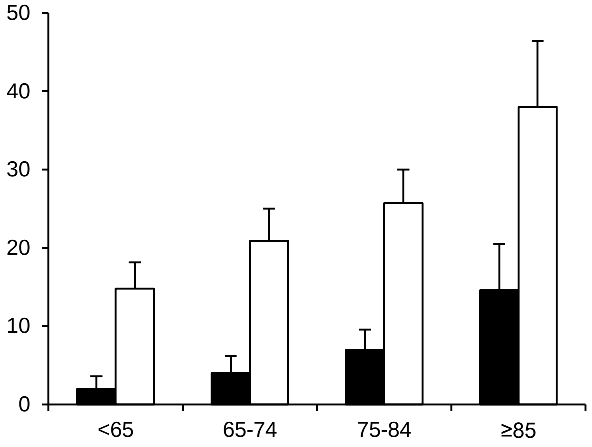


**MI cohort**

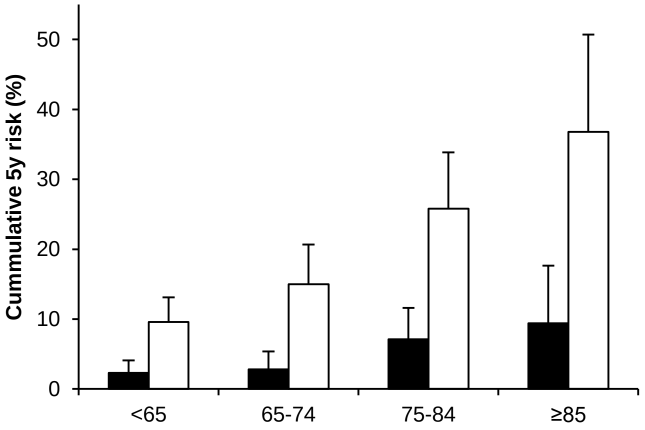


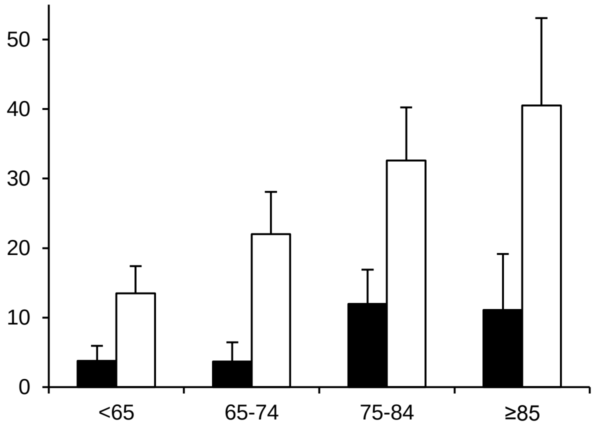


■ major bleed □ myocardial infarction or ischaemic stroke

**Web appendix 26. Sensitivity analyses censoring at a composite outcome of bleeding events or recurrent ischaemic events for the estimated age-specific 5-year risk of major bleeding attributable to antiplatelet treatment vs. ischaemic events prevented by antiplatelet treatment.**

(Patients with history of atrial fibrillation on antiplatelet treatment excluded;MI=myocardial infarction; data presented with 95%CI)

**Excluding recurrent ischaemic or bleeding events within 90 days post index events** | **All events included**

**Whole cohort**

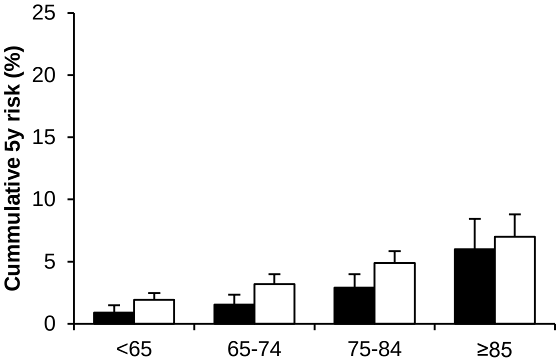


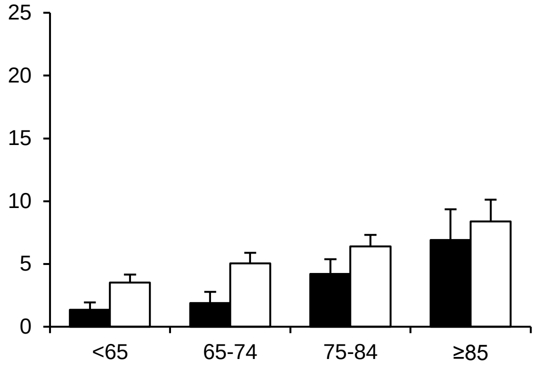


**Cerebrovascular cohort**

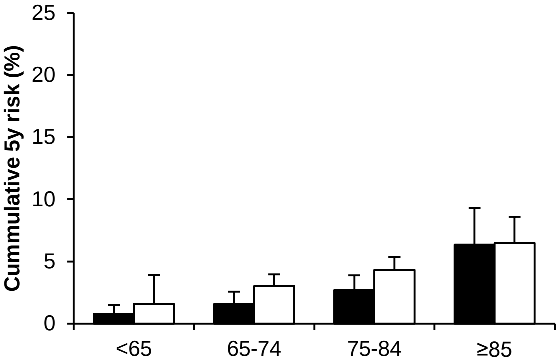


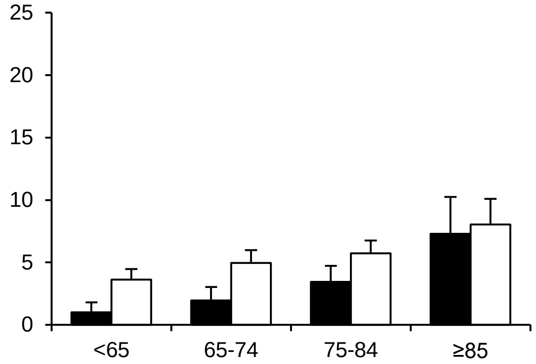


**MI cohort**

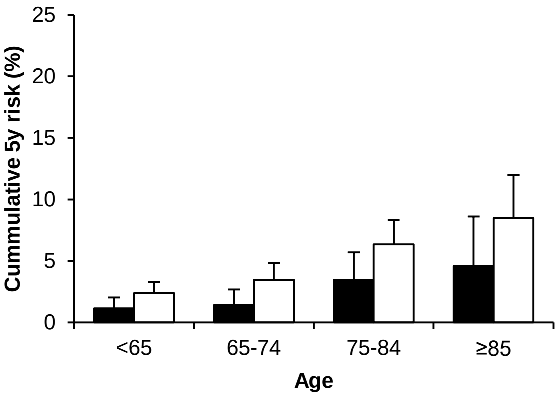


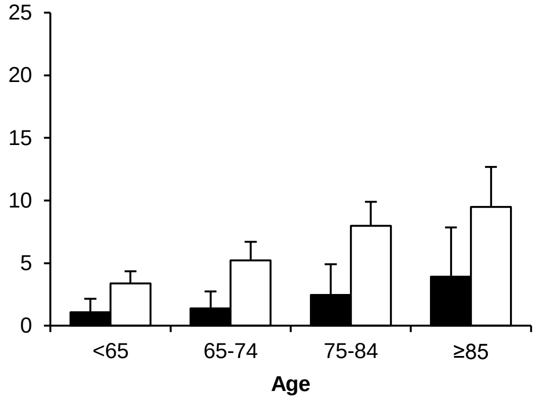


■ major bleed □ myocardial infarction, sudden cardiac death or non-fatal ischaemic stroke

**Web appendix 27. Estimated age-specific numbers-needed-to-treat (NNT) to prevent one upper-GI bleed based on different lengths of follow-up in patients on antiplatelet treatment for secondary prevention of vascular events.** (Based on the cumulative risks from the Kaplan-Meier curve, using the reported relative risk reduction of 0.26 from a recent systematic review and assuming the efficacy of PPI was similar at different ages and remained consistent with time)

**A. Any bleed**

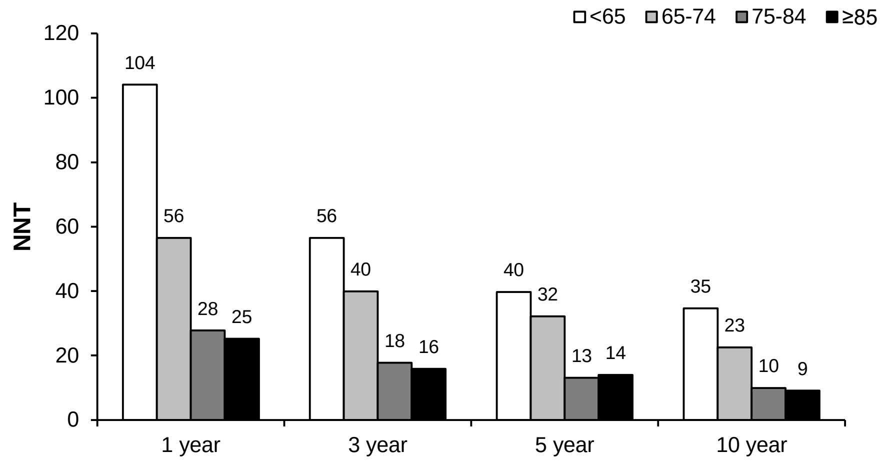


**B. Any major bleed**

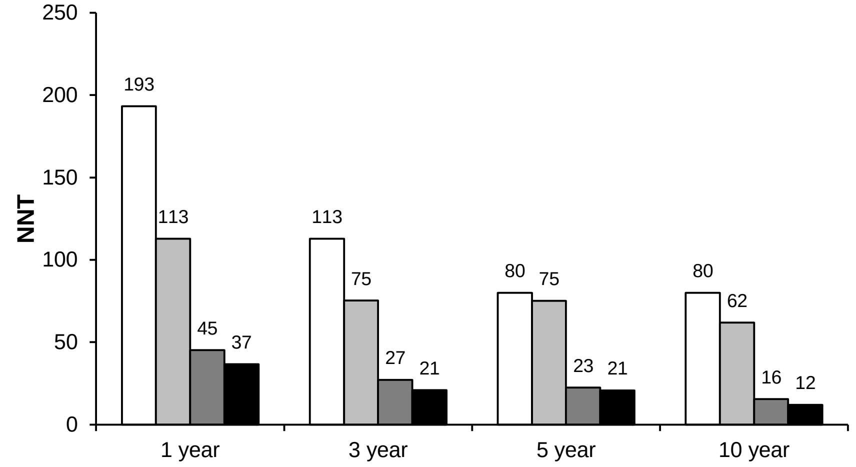


**C. Any disabling or fatal bleed**

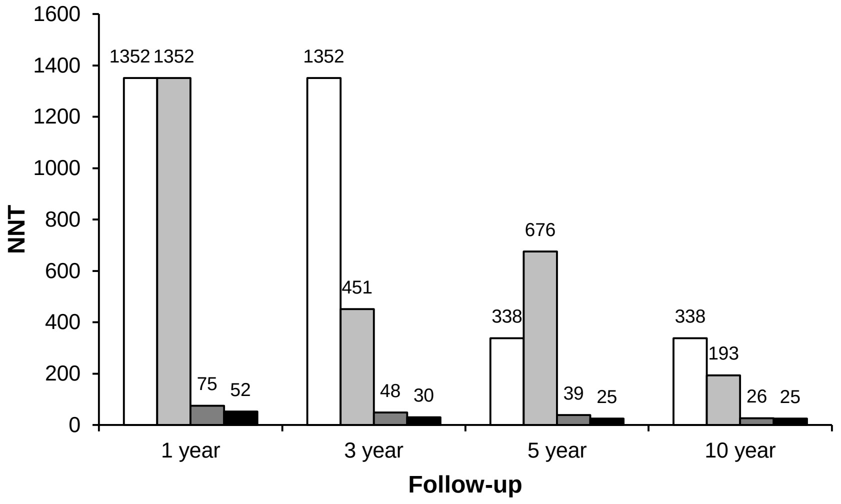

**Web appendix 28. Sensitivity analyses of the estimated age-specific numbers-needed-to-treat (NNT) to prevent one upper-GI bleed based on different lengths of follow-up in patients on antiplatelet treatment for secondary prevention of vascular events excluding patients with previous peptic ulcer or excluding patients on premorbid gastric protection agents.** (Based on the cumulative risks from the Kaplan-Meier curve, using the reported relative risk reduction of 0.26 from a recent systematic review and assuming the efficacy of PPI was similar at different ages and remained consistent with time)

**Excluding patients with previous peptic ulcer**

**Excluding patients on premorbid gastric protection**

**Any bleed**

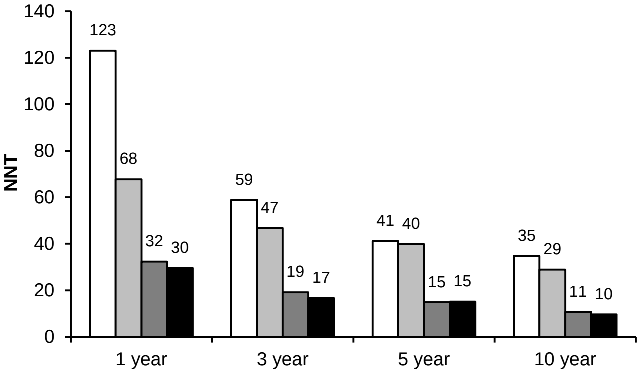


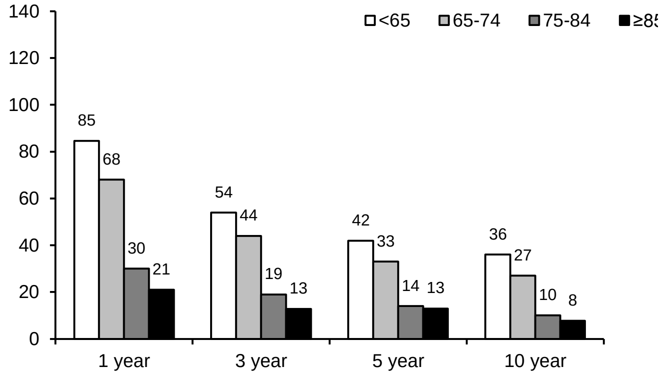


**Any major bleed**

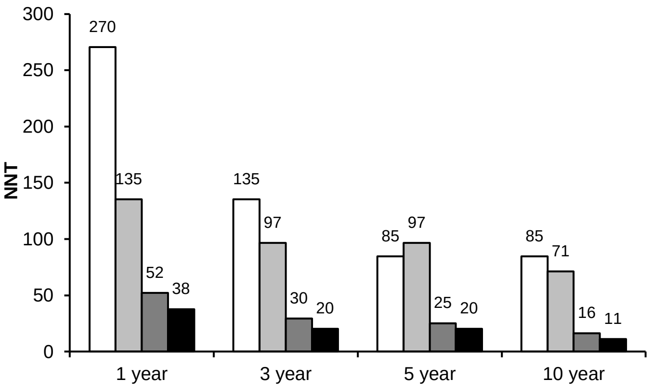


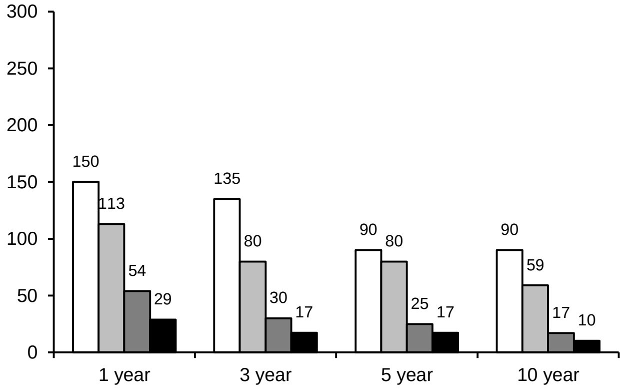


**Any disabling or fatal bleed**

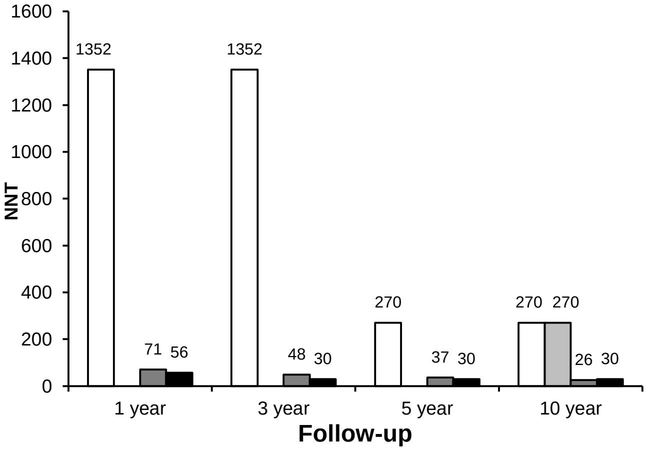


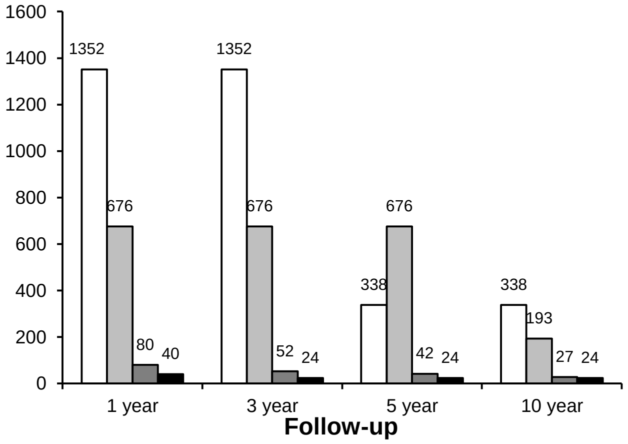

**Web appendix 29. References for PPI trials in the prevention of antiplatelet-associated upper-GI complications.**


e1. Lai KC, Lam SK, Chu KM, et al. Lansoprazole for the prevention of recurrences of ulcer complications from long-term low-dose aspirin use. New Engl J Med 2002; 346: 2033-8.

e2. Fujita T, Kutsumi H, Sanuki T, Hayakumo T, Azuma T. Adherence to the preventive strategies for nonsteroidal anti-inflammatory drug- or low-dose aspirin-induced gastrointestinal injuries. J Gastroenterol 2013; 48: 559-73.

e3. Scheiman JM, Devereaux PJ, Herlitz J, et al. Prevention of peptic ulcers with esomeprazole in patients at risk of ulcer development treated with low-dose acetylsalicylic acid: a randomised, controlled trial (OBERON). Heart 2011; 97: 797-802.

e4. Hsu PI, Lai KH, Liu CP. Esomeprazole with clopidogrel reduces peptic ulcer recurrence, compared with clopidogrel alone, in patients with atherosclerosis. Gastroenterology 2011; 140: 791-8.

e5. Yeomans N, Lanas A, Labenz J, et al. Efficacy of esomeprazole (20 mg once daily) for reducing the risk of gastroduodenal ulcers associated with continuous use of low-dose aspirin. Am J Gastroenterol 2008; 103: 2465-73.

e6. Bhatt DL, Cryer BL, Contant CF, et al. Clopidogrel with or without omeprazole in coronary artery disease. New Engl J Med 2010; 363: 1909-17.

e7. Ng FH, Wong SY, Lam KF, et al. Famotidine is inferior to pantoprazole in preventing recurrence of aspirin-related peptic ulcers or erosions. Gastroenterology 2010; 138: 82-8.

e8. Sugano K, Matsumoto Y, Itabashi T, et al. Lansoprazole for secondary prevention of gastric or duodenal ulcers associated with long-term low-dose aspirin therapy: results of a prospective, multicenter, double-blind, randomized, double-dummy, active-controlled trial. J Gastroenterol 2011; 46: 724-35.

e9. Sanuki T, Fujita T, Kutsumi H, et al. Rabeprazole reduces the recurrence risk of peptic ulcers associated with low-dose aspirin in patients with cardiovascular or cerebrovascular disease: a prospective randomized active-controlled trial. J Gastroenterol 2012; 47: 1186-97.

e10. Chan FK, Chung SC, Suen BY, et al. Preventing recurrent upper gastrointestinal bleeding in patients with Helicobacter pylori infection who are taking low-dose aspirin or naproxen. New Engl J Med 2001; 344: 967-73.

e11. Ng FH, Tunggal P, Chu WM, et al. Esomeprazole compared with famotidine in the prevention of upper gastrointestinal bleeding in patients with acute coronary syndrome or myocardial infarction. Am J Gastroenterol 2012; 107: 389-96.

**Web appendix 30. References for aspirin-controlled secondary prevention trials.**


e12. Fields WS, Lemak NA, Frankowski RF, Hardy RJ. Controlled Trial of Aspirin in Cerebral Ischemia. Stroke 1977;8:301-16.

e13. Farrell B, Godwin J, Richards S, Warlow C. The United-Kingdom Transient Ischemic Attack (Uk-Tia) Aspirin Trial - Final Results. J Neurol Neurosur Ps 1991;54:1044-54.

e14. Vangijn J, Algra A, Kappelle J, Koudstaal PJ, Vanlatum LJ, Grp DTTS. A Comparison of 2 Doses of Aspirin (30 Mg Vs 283 Mg a Day) in Patients after a Transient Ischemic Attack or Minor Ischemic Stroke. New Engl J Med 1991;325:1261-6.

e15. Norrving B, Elwin CE, Peterson B, et al. Swedish Aspirin Low-Dose Trial (Salt) of 75 Mg Aspirin as Secondary Prophylaxis after Cerebrovascular Ischemic Events. Lancet 1991;338:1345-9.

e16. Britton M, Helmers C, Samuelsson K. High-Dose Acetylsalicylic-Acid after Cerebral Infarction. Stroke 1987;18:325-34.

e17. Diener HC, Cunha L, Forbes C, Sivenius J, Smets P, Lowenthal A. European stroke prevention study .2. Dipyridamole and acetylsalicylic acid in the secondary prevention of stroke. J Neurol Sci 1996;143:1-13.

e18. Group ES, Halkes PH, van Gijn J, Kappelle LJ, Koudstaal PJ, Algra A. Aspirin plus dipyridamole versus aspirin alone after cerebral ischaemia of arterial origin (ESPRIT): randomised controlled trial. Lancet 2006;367:1665-73.

e19. Benavente OR, Hart RG, McClure LA, et al. Effects of Clopidogrel Added to Aspirin in Patients with Recent Lacunar Stroke. New Engl J Med 2012;367:817-25.

e20. Gent M, Beaumont D, Blanchard J, et al. A randomised, blinded, trial of clopidogrel versus aspirin in patients at risk of ischaemic events (CAPRIE). Lancet 1996;348:1329-39.

e21. Mohr JP, Thompson JLP, Lazar RM, et al. A comparison of warfarin and aspirin for the prevention of recurrent ischemic stroke. New Engl J Med 2001;345:1444-51.

e22. Chimowitz MI, Lynn MJ, Howlett-Smith H, et al. Comparison of warfarin and aspirin for symptomatic intracranial arterial stenosis. New Engl J Med 2005;352:1305-16.

e23. Franke CL, Koehler PJJ, Gorter JW, et al. A randomized trial of anticoagulants versus aspirin after cerebral ischemia of presumed arterial origin. Ann Neurol 1997;42:857-65.

e24. Bousser MG, Eschwege E, Haguenau M, et al. "AICLA" controlled trial of aspirin and dipyridamole in the secondary prevention of athero-thrombotic cerebral ischemia. Stroke 1983;14:5-14.

e25. Fields WS, Lemak NA, Frankowski RF, Hardy RJ. Controlled Trial of Aspirin in Cerebral Ischemia .2. Surgical Group. Stroke 1978;9:309-19.

e26. Elwood PC, Cochrane AL, Burr ML, et al. A randomized controlled trial of acetyl salicylic acid in the secondary prevention of mortality from myocardial infarction. Br Med J 1974;1:436-40.

e27. Elwood PC, Sweetnam PM. Aspirin and secondary mortality after myocardial infarction. Lancet 1979;2:1313-15.

e28. Persantine-Aspirin Reinfarction Study (PARIS) Research Group. Persantine-Aspirin Reinfarction Study. Design, methods and baseline results. Circulation 1980;62:449-61.

e29. Aspirin Myocardial Infarction Study (AMIS) Research Group. A randomized, controlled trial of aspirin in persons recovered from myocardial infarction. JAMA 1980;243:661-9.

e30. Coronary Drug Profect (CDP) Research Group. Aspirin in coronary heart disease. The Coronary Drug Project Research Group. J Chronic Dis 1976;29:625-42.

e31. Breddin K, Loew D, Lechner K, Uberla K, Walter E. Secondary prevention of myocardial infarction: a comparison of acetylsalicylic acid, placebo and phenprocoumon. Haemostasis 1980;9:325-44.

e32. Reuther R, Dorndorf W. Aspirin in patients with cerebral ischemia and normal angiograms or non-surgical lesions. In: Acetylsalicylic acid in cerebral ischemia and coronary heart disease. Breddin K, Dorndorf W, Loew D, Marx R, ef. Stuttgart: Schattauer; 1978:97-106.

e33. Canadian Cooperative Study Group. A randomized trial of aspirin and sulfinpyrazone in threatened stroke. The Canadian Cooperative Study Group. New Engl J Med 1978;299:53-9.

e34. Guiraud-Chaumeil B, Rascol A, David J, Boneu B, Clanet M, Bierme R. Prevention des recidives des accidents vasculaires cerebraux ischemiques par les anti-agregants plaquettaires. Rev Neurol (Paris) 1982; 138: 367-85.

e35. Sorensen PS, Pedersen H, Marquardsen J, et al. Acetylsalicylic acid in the prevention of stroke in patients with reversible cerebral ischemic attacks. A Danish cooperative study. Stroke 1983;14:15-22.

e36. Boysen G, Sorensen PS, Juhler M, Andersen AR, Boas J, Olsen JS, et al. Danish very-low-dose aspirin after carotid endarterectomy trial. Stroke 1998; 19:1211-5.

**Web appendix Methods - OXVASC methodology**

**Study population**

The Oxford Vascular Study (OXVASC) is a prospective, population-based cohort study of all incident acute vascular events in all territories (transient ischaemic attack, stroke, acute coronary and peripheral vascular events).

The study population consisted of all 92,728 individuals, irrespective of age, registered with 100 general practitioners (GPs) in nine general practices in Oxfordshire, UK. In the UK, general practices provide primary health care for registered individuals and hold a lifelong record of all medical consultations (from the National Health Service [NHS] and private health care), and details of treatments, blood pressure, and investigations. In Oxfordshire, an estimated 97% of the true residential population is registered with a general practice, with most non-registered individuals being young adults. All participating practices held accurate age-sex patient registers, and allowed regular searches of their computerised diagnostic coding systems. The practices had all collaborated on a previous population-based study, for which they were originally selected to be representative of the urban and rural mix and the deprivation range of Oxfordshire as a whole.[1] Based on the index of multiple deprivation (IMD), the population was less deprived than the rest of England, but had a broad range of deprivation.

The OXVASC population is 94% white people, 3% Asian, 2% Chinese, and 1% Afro-Caribbean.[2] The proportion of whites is similar to that of the UK as a whole (88% white) and to many other western countries ((Australia - 90%; France - 91%; Germany - 93.9%).

**Case ascertainment**

After a 3-month pilot study, the study started on April 1, 2002, and is ongoing. Ascertainment combined prospective daily searches for acute events (hot pursuit) and retrospective searches of hospital-care and primary-care administrative and diagnostic coding data (cold pursuit).

Hot pursuit was based on:

1) A daily (weekdays only), urgent open-access "TIA clinic" to which participating general practitioners (GPs) and the local accident and emergency department (A&E) send all individuals with suspected TIA or stroke whom they would not normally admit to hospital, with alternative on-call review provision at weekends. Patients too frail to attend are assessed at their residence by a study nurse or doctor.
2) Daily searches and case note review of admissions to the Emergency Assessment Unit, Medical Short Stay Unit, Coronary Care Unit and Cardiothoracic Critical Care Unit, Cardiology, Cardiothoracic, and Vascular Surgery wards, Acute Stroke Unit, Neurology ward and all other general wards when indicated.
3) Daily searches of the local A&E and eye hospital attendance registers.
4) Daily identification via the Bereavement Office of patients dead on arrival at hospital or who died soon after.
5) Daily searches of lists of all patients from the study population in whom a troponin-I level had been requested.
6) Daily assessment of all patients undergoing diagnostic coronary, carotid and peripheral angiography, angioplasty, stenting or vascular surgical procedures in any territory to identify both total burden of vascular invention and any potential missed prior acute events.

Cold pursuit procedures were:

1) Frequent visits to the study practices and monthly searches of practice diagnostic codes.
2) Monthly practice-specific list of all patients admitted to all acute and community NHS hospitals.
3) Monthly listings of all referrals for brain or carotid imaging studies performed in local hospitals.
4) Monthly reviews of all death certificates and coroners reports to review out-of-hospital deaths.
5) Practice-specific listings of all ICD-10 death codes from the local Department of Public Health.

Patients found on GP practice searches who have an event whilst temporarily out of Oxfordshire are included, but visitors who were not registered with one of the study practices are excluded. A study clinician assessed patients as soon as possible after the event in the hospital or at home. Informed consent was sought, if possible, or assent was obtained from a relative.

Data is collected using event-specific forms, for TIA and stroke, acute coronary syndrome or acute peripheral vascular events. Standardised clinical history and cardiovascular examination are recorded. Information recorded from the patient, their hospital records and their general practice records includes details of the clinical event,

medication, past medical history, all investigations relevant to their admission (including blood results, electrocardiography, brain imaging and vascular imaging-duplex ultrasonography, CT-angiography, MR-angiography or DSA) and all interventions occurring subsequent to the event.

If a patient died before assessment, we obtained an eyewitness account of the clinical event and reviewed any relevant records. If death occurred outside the hospital or before investigation, the autopsy result was reviewed. Clinical details are sought from primary care physicians or other clinicians on all deaths of possible vascular aetiology.

All surviving patients are followed-up face-to-face at 1, 6, 12, 60 and 120 months after the initial event by a research nurse or physician and all recurrent vascular events were recorded together with the relevant clinical details and investigations. If face-to-face follow up is not possible, telephone follow-up is performed or enabled via the general practitioner. All recurrent vascular events that presented to medical attention would also be identified acutely by ongoing daily case ascertainment within OXVASC. If a recurrent vascular event was suspected at a follow-up visit or referred by the GPs to clinic or admitted, the patient was re-assessed and investigated by a study physician.

**Definition of diagnosis**

Although new definitions for stroke and TIA have been suggested recently,[2,3] in order to enable comparison with previous studies, the classic definitions of TIA and stroke are used throughout.[4] A stroke is defined as rapidly developing clinical symptoms and/or signs of focal, and at time global (applied to patients in deep coma and to those with subarachnoid haemorrhage), loss of brain function, with symptoms lasting more than 24 hours or leading to death, with no apparent cause other than that of vascular origin.[4] A TIA is an acute loss of focal brain or monocular function with symptoms lasting less than 24 hours and which is thought to be caused by inadequate cerebral or ocular blood supply as a result of arterial thrombosis, low flow or embolism associated with arterial, cardiac or haematological disease.[4] All diagnoses were reviewed by a senior neurologist (PMR). With the high rate (97%) of imaging or autopsy in OXVASC, strokes of unknown type were coded as ischaemic.

Acute coronary events are defined using published criteria[5] based on available history, electrocardiography (ECG) findings, cardiac biomarkers (mainly troponin I), and autopsy or death certificate. Non-ST elevation (NSTEMI) and ST-elevation myocardial infarction (STEMI) are defined using standard criteria.[6] Sudden cardiac deaths are coded according to recent recommendations for epidemiological studies,[5] and required a definite history of preceding symptoms consistent with acute coronary ischaemia, or post-mortem evidence of either significant coronary atherosclerosis or acute thrombosis, or a documented myocardial infarction during the previous 28 days.[5] Sudden deaths were coded as probable cardiac deaths in the absence of the above characteristics if the person had a past history of ischaemic heart disease.

**References**


1. Bamford J, Sandercock P, Dennis M, Burn J, Warlow C. A prospective study of acute cerebrovascular disease in the community: the Oxfordshire Community Stroke Project--1981-86. 2. Incidence, case fatality rates and overall outcome at one year of cerebral infarction, primary intracerebral and subarachnoid haemorrhage. J Neurol Neurosurg Psychiatry 1990;53:16-22.
2. Easton JD, Saver JL, Albers GW, et al. Definition and evaluation of transient ischemic attack: a scientific statement for healthcare professionals from the American Heart Association/American Stroke Association Stroke Council; Council on Cardiovascular Surgery and Anesthesia; Council on Cardiovascular Radiology and Intervention; Council on Cardiovascular Nursing; and the Interdisciplinary Council on Peripheral Vascular Disease. The American Academy of Neurology affirms the value of this statement as an educational tool for neurologists. Stroke 2009;40:2276-2293.
3. Sacco RL, Kasner SE, Broderick JP, et al. An updated definition of stroke for the 21st century: a statement for healthcare professionals from the American Heart Association/American Stroke Association. Stroke 2013;44:2064-2089.
4. Hatano S. Experience from a multicentre stroke register: a preliminary report. Bulletin of the World Health Organization 1976;54:541-553.
5. Luepker RV, Apple FS, Christenson RH, et al. Case definitions for acute coronary heart disease in epidemiology and clinical research studies: a statement from the AHA Council on Epidemiology and Prevention; AHA Statistics Committee; World Heart Federation Council on Epidemiology and Prevention; the European Society of Cardiology Working Group on Epidemiology and Prevention; Centers for Disease Control and Prevention; and the National Heart, Lung, and Blood Institute. Circulation 2003;108:2543-2549.
6. Alpert JS, Thygesen K, Antman E, Bassand JP. Myocardial infarction redefined--a consensus document of The Joint European Society of Cardiology/American College of Cardiology Committee for the redefinition of myocardial infarction. J Am Coll Cardiol 2000;36:959-969.

**Web appendix Methods – Additional details of statistical analyses**

**Risk factors included in the analyses to determine predictors of major bleeding and major upper-GI bleeding**

Age, sex, body weight, history of vascular disease, known hypertension, diabetes, hyperlipidaemia, smoking, alcohol use, baseline anaemia, history of cancer, chronic liver disease, renal failure, atrial fibrillation, chronic heart failure, history of peptic ulcer, premorbid antiplatelet use, premorbid use of gastric protection and dual antiplatelet therapy post event.

**Calculation age-specific antiplatelet treatment attributable risks and benefit**

To compare the long-term risk of major bleeding likely to be attributable to antiplatelet treatment with the estimated benefit in terms of ischaemic events likely to have been prevented, we used estimates of relative risk derived from previous meta-analyses of trials of aspirin use versus control in secondary prevention of vascular events:[1] i.e. a 20% relative reduction in ischaemic events and a doubling in major bleeds. Aspirin-preventable vascular events included myocardial infarction, sudden cardiac death and non-fatal ischaemic stroke. This analysis was stratified by age, assuming the relative effects of aspirin were independent of age.

**Sensitivity analyses**

**1. Age-specific bleeding risks**

Primary analyses included all bleeding events irrespective of antiplatelet medication change during follow-up. However, sensitivity analyses were also performed for bleeding risk censoring patients when antiplatelet medication was stopped, and with exclusion of bleeds that occurred during periods of dual treatment with aspirin plus clopidogrel.

**2. Comparing the long-term risks of major bleeding and risks of recurrent ischaemic events**

Primary analyses included all events during follow-up. However sensitivity analyses were also performed excluding bleeding and ischaemic events <90 days after the index event or censoring at the time of either a first major bleed or a first ischaemic event.

**3. Estimated NNT for PPI use**

Primary analyses included all patients. However, sensitivity analyses were also performed for NNTs for PPI use in patients without history of peptic ulcer and for patients not premorbidly on any gastric protection agents.

**References**

1. Antithrombotic Trialists C, Baigent C, Blackwell L, et al. Aspirin in the primary and secondary prevention of vascular disease: collaborative meta-analysis of individual participant data from randomised trials. *Lancet* 2009; 373: 1849-60.

**Web appendix Methods - Criteria and risk scores used in the study**

**CURE criteria**

Major bleeding
- Life-threatening (fatal, intracranial, requiring surgical intervention, results in substantial hypotension requiring the use of intravenous inotropic agents)
  - ✓ Hemoglobin decrease ≥5 g/dL or required ≥4 U of blood
- Other major bleeding
  - ✓ Transfusion of 2–3 U, intraocular

Non-major bleeding
- Led to discontinuation of study drug

**Reference**

Yusuf S, Zhao F, Mehta SR, et al. Effects of clopidogrel in addition to aspirin in patients with acute coronary syndromes without ST-segment elevation. N Engl J Med 2001; 345: 494-502.

**ABCD$^2$ score for risk stratification of transient ischaemic attack (TIA)**

| Factor | Points |
|---|---|
| Age≥60 | 1 |
| Blood pressure≥140/90mmHg | 1 |
| Clinical features | |
| Unilateral weakness | 2 |
| Speech disturbance without weakness | 1 |
| Other symptoms | 0 |
| Duration of symptoms | |
| <10min | 0 |
| 10-59min | 1 |
| ≥60min | 2 |
| History of diabetes | 1 |

**Reference**

Johnston SC, Rothwell PM, Nguyen-Huynh MN, et al. Validation and refinement of scores to predict very early stroke risk after transient ischaemic attack. Lancet 2007; 369: 283–92.

**Reduction of Atherothrombosis for Continued Health (REACH) registry score**

| Factor | Points | | | |
|---|---|---|---|---|
| Age/years | 45-54 | 55-64 | 65-74 | 75+ |
| | 0 | 2 | 4 | 6 |
| Peripheral arterial disease | No | Yes | | |
| | 0 | 1 | | |
| Congestive heart failure | No | Yes | | |
| | 0 | 2 | | |
| Diabetes | No | Yes | | |
| | 0 | 1 | | |
| Hypercholesterolaemia | No | Yes | | |
| | 1 | 0 | | |
| Hypertension | No | Yes | | |
| | 0 | 2 | | |
| Smoking | Never | Former | Current | |
| | 0 | 1 | 2 | |
| Antiplatelet agents | None | Aspirin | Other | Both |
| | 0 | 1 | 2 | 4 |
| Oral anticoagulants | No | Yes | | |
| | 0 | 4 | | |

**Reference**

Ducrocq G, Wallace JS, Baron G, et al. Risk score to predict serious bleeding in stable outpatients with or at risk of atherothrombosis. Eur Heart J 2010; 31: 1257-65.

**Web appendix Additional discussion of PPI related harms**

We did not attempt to estimate the number-needed-to-harm in relation to potential adverse effects of long-term PPI use. In the absence of large randomised trials of long-term treatment, reliable estimation of any hazard is difficult. Although the clinical significance of the pharmacodynamics interaction of some PPIs and clopidogrel is unclear,[1] there is some evidence of a short-term risk of respiratory infection (approx. 2 per 1000-person-years),[1] but there is uncertainty about a causal link with PPI use,[2] and there is no evidence of any increase in relative hazard with age.[3] However, PPI use does reduce magnesium levels, which requires screening at baseline and during follow-up,[4] and might be more problematic in patients on diuretics. Other rare adverse effects include an increased risk of C. Difficile infection.[1]

For patients aged less than 75 years, given the relatively high NNT, and current concern about complications of long-term PPI use, the benefit of routine co-prescription is less clear. It is also unclear whether a PPI should be started when patients who have been well on long-term antiplatelet treatment reach age 75 years. Consistent with our previous findings,[5] we showed that the overall bleeding risk on antiplatelet treatment at younger ages seemed to decrease over long-term follow-up, either because high-risk patients stop treatment, go on to PPI, or possibly due to adaption to local effects in the stomach with chronic use.[6] However, in our study, 87% patients were still on antiplatelet treatment after 5 years and only about one third were on a PPI or H2-AR.

**References**


1. Moayyedi P, Leontiadis GI. The risks of PPI therapy. *Nat Rev Gastroenterol Hepatol* 2012; 9: 132-9.
2. Othman F, Crooks CJ, Card TR. Community acquired pneumonia incidence before and after proton pump inhibitor prescription: population based study. *BMJ* 2016; 355: i5813.
3. Lambert AA, Lam JO, Paik JJ, Ugarte-Gil C, Drummond MB, Crowell TA. Risk of community-acquired pneumonia with outpatient proton-pump inhibitor therapy: a systematic review and meta-analysis. *PLoS One 2015*; doi: 10.1371/journal.pone.0128004.
4. Park CH, Kim EH, Roh YH, Kim HY, Lee SK. The association between the use of proton pump inhibitors and the risk of hypomagnesemia: a systematic review and meta-analysis. *PLoS One 2014*; 9: doi: 10.1371/journal.pone.0112558
5. Rothwell PM, Price JF, Fowkes FG, et al. Short-term effects of daily aspirin on cancer incidence, mortality, and non-vascular death: analysis of the time course of risks and benefits in 51 randomised controlled trials. Lancet 2012; 379: 1602-12.
6. Kawai T, Yamagishi T, Goto S. Circadian variations of gastrointestinal mucosal damage detected with transnasal endoscopy in apparently healthy subjects treated with low-dose aspirin (ASA) for a short period. J Atheroscler Thromb 2009; 16: 155-63.
